# Genome-wide analysis of the *Cannabis sativa* cytochrome P450 monooxygenase superfamily and uncovering candidate genes for improved herbicide tolerance

**DOI:** 10.3389/fpls.2024.1490036

**Published:** 2024-11-07

**Authors:** Navneet Kaur, Awadhesh Kumar Verma, Madhuri Girdhar, Anil Kumar, Maqsood A. Siddiqui, Abdulaziz A. Al-Khedhairy, Tabarak Malik, Anand Mohan

**Affiliations:** ^1^ School of Bioengineering and Biosciences, Lovely Professional University, Phagwara, Punjab, India; ^2^ Division of Research and Development, School of Bioengineering and Biosciences, Lovely Professional University, Phagwara, Punjab, India; ^3^ Gene Regulation Laboratory, National Institute of Immunology, New Delhi, India; ^4^ Chair for DNA Research, Department of Zoology, College of Science, King Saud University, Riyadh, Saudi Arabia; ^5^ Department of Biomedical Sciences, Institute of Health, Jimma University, Jimma, Ethiopia

**Keywords:** cytochrome P450, *Cannabis sativa*, herbicide tolerance, ALS herbicides, molecular docking, homology modeling

## Abstract

*Cannabis sativa* is an economically important crop, yet weed management remains a significant challenge due to limited herbicide options. Cytochrome P450 enzymes play crucial roles in plant metabolism, including herbicide detoxification. This study aimed to identify and characterize the CYP gene family in Cannabis and investigate their potential role in herbicide metabolism. We identified 225 CYP proteins encoded by 221 genes in the Cannabis genome, classified into 9 clans and 47 families. The majority of CsCYPs were predicted to be located in endomembrane system and chromosomal mapping revealed that they were present in all the chromosomes. Motif and gene structure analysis supported the results from phylogenetic analysis. The gene duplication analysis results showed that tandem duplication plays a pivotal role in evolutionary expansion of CsCYP superfamily. Promoter analysis revealed various cis-acting elements involved in stress, light, hormone and development responses. Molecular docking simulations identified several CsCYPs with strong binding affinities to ALS-inhibiting herbicides, particularly bispyribac-sodium, propoxycarbazone-sodium, and pyriftalid. CsCYP_215, CsCYP_213, CsCYP_217 and CsCYP_14 emerged as promising candidates for herbicide metabolism. Analysis of binding site residues revealed the importance of hydrophobic and aromatic interactions in herbicide binding. This study provides the first comprehensive characterization of the CYP gene family in *C. sativa* and offers new insights into their potential roles in herbicide metabolism. The identification of promising herbicide-metabolizing CYP candidates opens new avenues for developing herbicide-tolerant Cannabis varieties, potentially addressing key challenges in weed management and crop productivity.

## 1 Introduction

*Cannabis sativa* is a multipurpose crop that has been cultivated for centuries across various geographies for applications ranging from medicinal and wellness products to industrial fibers and textiles (Salentijn et al., 2015; Baldini et al., 2018). Its production and demand have risen substantially in recent years owing to the progressive legalization of its cultivation and use across numerous countries (Cherney and Small, 2016; Hillebrands et al., 2021). As of 2021, medical and/or recreational cannabis has been fully or partially legalized across 64 countries, while hemp cultivation is currently permitted across 30 countries in Asia, Europe, and America (Kaur and Kander, 2023; United Nations Office on Drugs and Crime, 2023).

However, multiple environmental and agronomic factors limit Cannabis productivity and sustainability, especially biotic and abiotic stresses. Weed competition is a major biological factor that hinders growth by competing for space, sunlight, soil moisture, and nutrients (Williams and Mundell, 2016; Schluttenhofer and Yuan, 2017; Flessner et al., 2020). There is a scarcity of published research on weed management approaches tailored to Cannabis crops. Available literature indicates that increasing crop density by raising hemp seeding rates can suppress weeds to some extent. However, there remains an urgent need to elucidate and implement effective large-scale weed management solutions to enhance Cannabis productivity (Sandler and Gibson, 2019).

Chemical weed control using herbicides is the most widely adopted technology enabling efficient, economical, and scalable weed control in global agriculture (Gianessi and Reigner, 2007; Haggblade et al., 2017). Unfortunately, research reveals that hemp growth and development is sensitive to many commercially available herbicides. Field experiments testing 55 common herbicides found that most triggered over 50% biomass reduction in hemp due to phytotoxic effects when applied at typical field application rates (Ortmeier-Clarke et al., 2022). Simultaneously, indiscriminate long-term use of herbicides is driving widespread evolution of resistance among different weeds. To date, weeds have developed resistance towards twenty-one out of thirty-one known herbicide action sites and over 165 various herbicides through a combination of target-site and non-target-based mechanisms. Continued change in the use of different modes of action herbicides is critical to restrict the further evolution of herbicide resistance in weeds (Ofosu et al., 2023). Hence, there is a need to enhance tolerance within Cannabis for its wide growth potential and application to compete successfully with weeds.

Among available commercial herbicides, acetolactate synthase (ALS) inhibitors have a major share valued at ~$US30 billion per year in the weed management market (Garcia et al., 2017). The enzyme ALS plays a crucial role in plants by initiating the production of essential branched-chain amino acids: isoleucine, leucine, and valine. Herbicides that target ALS prevent the synthesis of these vital amino acids, resulting in the plant’s inability to grow and eventually leading to its death. There are six distinct chemical groups that make up the ALS inhibitor class of herbicides. These include sulfonylureas, imidazolinones, triazolopyrimidines, pyrimidinyl-benzoates, triazolinones, and sulfonanilides (HRAC, 2024). Since their introduction in the early 1980s, ALS inhibitors have gained widespread popularity in agriculture worldwide due to their effectiveness against a broad range of weeds, very low use rates, their safety for crops, and their low toxicity to mammals (Gutteridge et al., 2011; Yu and Powles, 2014). Considering their dominant worldwide market share, ALS-inhibiting herbicide chemistries will be the primary focus for the characterization of herbicide metabolism pathways in Cannabis P450s.

Cytochrome P450 monooxygenases (P450s) are heme-containing enzymes that catalyze the oxidation of some herbicides and thus govern herbicide selectivity in crops (Dimaano and Iwakami, 2021). While P450 research in plants has revealed key aspects of their herbicide metabolism capacity, several knowledge gaps persist. To begin with, plant cytochrome P450 enzymes comprise one of the most extensive protein families found in plants. These enzymes frequently exhibit overlapping roles and promiscuous functions (Abdollahi et al., 2021; Werck-Reichhart, 2023). As a result, discovering which specific P450 enables herbicide tolerance is challenging, especially in non-model crops with minimal genomic resources. Secondly, while P450s demonstrate some substrate selectivity, there is also a significant crossover - P450s from different gene families, that can metabolize similar herbicides. Individual P450s also metabolise multiple herbicides from related or distinct chemical classes as substrates (Dimaano and Iwakami, 2021). Thus, it is important to pinpoint which P450s drive tolerance to specific herbicides. P450s involved in endogenous biosynthetic pathways, such as those for fatty acids and secondary metabolites also demonstrate an affinity for xenobiotic compounds like herbicides (Abdollahi et al., 2021).

The evolution of sequencing technologies has led to a surge in the availability of plant genomic and transcriptomic data, expanding the possibilities for research in this field. In recent years, scientists have identified numerous genes belonging to the P450 family in various plant species such as *Arabidopsis thaliana* (Xu et al., 2001), *Oryza sativa* (Wei and Chen, 2018), *Nelumbo nucifera* (Nelson and Schuler, 2013), *Glycine max* (Guttikonda et al., 2010), *Linum usitatissimum* L (Babu et al., 2013), *Morus notabilis* (Ma et al., 2014) and *Taxus chinensis* (Liao et al., 2017). However, reports on this gene family in *C. sativa* have been limited. The elucidation of the *C. sativa* genome sequence and availability of several annotated P450 sequences now permits a systematic, genome-wide study to analyse the cytochrome p450 gene family and pinpoint putative herbicide-metabolizing cytochrome P450 gene candidates. *In-silico* approaches like homology modeling and molecular docking simulations offer the flexibility to investigate their substrate specificities by probing their binding with various commercial herbicides. Past research in rice showed that overexpression of the P450 gene CYP81Q32 boosted tolerance to the herbicide mesosulfuron-methyl, demonstrating the importance of transcriptional elements in modulating herbicide metabolism (Wang et al., 2023b). Hence, decoding the cis-regulatory elements governing P450 expression is also crucial.

In summary, the use of herbicides is integral to meet rising demands and preventing losses to weeds, while curbing the evolution of resistance. Tailoring P450-mediated herbicide metabolism in Cannabis to expand its tolerance spectrum could sustain productivity when facing weed competition.

Therefore, the main focus of this research is to uncover the cytochrome P450s in *C. sativa*. This study comprehensively analyzed the whole genome to identify the *C. sativa* P450 gene family and studied the physicochemical properties, structural functions, evolutionary relationships, chromosomal locations, promoter characteristics, and collinearity associations of the identified proteins using bioinformatics tools. It also identified and modeled putative herbicide-metabolizing P450 protein candidates via comparative genomics and simulated binding with various commercial herbicides to pinpoint substrate specificity.

This investigation provides a theoretical foundation for understanding the functions of CsCYP (*C. sativa* cytochrome p450) genes and facilitates molecular breeding efforts in *C. sativa*. These fundamental insights can pave the way for targeted improvement of *C. sativa* to withstand a wider spectrum of herbicides. By sustaining productivity despite intense weed pressure, this work will aid in unlocking the full genetic potential of this multipurpose crop to meet rising global demands across industries.

## 2 Materials and methods

### 2.1 Mining and characterisation of cytochrome p450s in *C. sativa*

The reference proteome data of *C. sativa* was downloaded from National Center for Biotechnology Information website (NCBI, GCF_029168945.1) (accessed on 10 April 2024). The CsCYPs were identified according to the following procedure. Firstly, all of the *Arabidopsis thaliana* p450 protein sequences were downloaded from the Cytochrome P450 Homepage (accessed on 10 April 2024) and further aligned to construct the Hidden Markov Model profile (Krejci et al., 2016) using hmmbuild in HMMER3.4 software. Moreover, HMM profile of P450 domain (PF00067) was obtained from Interpro Pfam database (accessed on 10 April 2024) (El-Gebali et al., 2019). Consequently, Hmmsearch was performed against the local database with a threshold of e-value < 1e-10. In parallel, BLASTP searchwas performed against the *C. sativa* proteome database using all of the *Arabidopsis thaliana* P450s. Subsequently, NCBI-Conserved Domain Database (accessed on 24 April 2024) was used to select sequences containing the P450 protein domain (Wang et al., 2023a). After the screening, sequence alignment and analysis were carried out to remove redundant sequences.

### 2.2 Physicochemical characterisation, subcellular localisation prediction and chromosomal location analysis

To further characterise these identified proteins, various parameters such as, theoretical isoelectric points (pIs), molecular weights (MWs), instability index, aliphatic index, and grand average of hydropathicity of these proteins were estimated by Protparam tool of ExPASy Server (accessed on 29 April 2024) (Gasteiger et al., 2005). Subcellular localisation of the identified p450 proteins were predicted using BUSCA web server (accessed on 10 May 2024) (Savojardo et al., 2018). The gene location information was obtained from GFF file downloaded from NCBI (accessed on 10 April 2024). Finally, TBtools-II (Chen et al., 2020) was used to visualise CsP450 genes on the chromosomes and named them according to their positions on the chromosomes.

### 2.3 Phylogenetic analysis of the CsCYP proteins

To investigate the phylogenetic relationships among the CYPs, multiple sequence alignment of the *Arabidopsis thaliana* P450s (AtCYPs) and CsCYPs was performed using ClustalW with default parameters (Thompson et al., 1994). A representative from each plant P450 family across various species was also chosen for sequence alignment and phylogenetic tree construction. ClustalW was employed for multiple sequence alignment of P450 protein sequences between *C. sativa* (225), *Arabidopsis thaliana* (72), *Solanum tuberosum* (4), *Oryza sativa* (2), *Populus trichocarpa* (1) and *Solanum Lycopersicon* (1) using default parameters. The phylogenetic tree was constructed using the maximum likelihood method in IQ-TREE web server (accessed on 3 May 2024) (Nguyen et al., 2015), and the appropriate model was selected utilizing the ModelFinder method (accessed on 3 May 2024) (Kalyaanamoorthy et al., 2017). The ML tree was built with 5000 ultrafast bootstrap replications (Hoang et al., 2018) and visualised using iTOL.

### 2.4 Gene structure and conserved motif identification

The gene structure information was obtained from GFF file downloaded from NCBI. The exon-intron structure along with p450 domains was visualised using the “Gene structure view” tool in Tbtools-II software (Chen et al., 2020). The conserved motifs in CsCYP proteins were predicted using MEME online website (accessed on 27 April 2024) (Bailey et al., 2015). The maximum number of motifs was set to 15 and the rest of the parameters were set to default (Liu et al., 2023). The results were visualised using TBtools-II (Chen et al., 2020).

### 2.5 Analysis of CsCYPs cis-acting regulatory elements

To analyze promoter elements within the CsCYPs, 2000 bp upstream promoter region was extracted using TBtools-II software for all the p450 genes. These were submitted to the PlantCARE website (accessed on 28 April 2024) (Lescot et al., 2002), for the identification of cis-regulatory elements. Visualization of the obtained data was performed using Tbtools-II (Chen et al., 2020).

### 2.6 Collinearity and synteny analysis

The Multiple Collinearity Scan tool kit (MCScanX) (Wang et al., 2012) was utilised to examine the gene duplication events and collinearity relationship amongst CsCYPs, using default parameters. Density profile was produced by tbtools. Syntenic block, duplicated CYP gene pairs (tandem and segmental duplications) and gene density were visualized using circos in tbtools. Using the Simple Ka/Ks Calculator function included in TBtools, the non-synonymous (Ka) and synonymous (Ks) substitution rates of duplicated CYP gene pairs were determined. Furthermore, TBtools was used to map the duplicated CsCYPs genes to the *C. sativa* genome’s chromosomes. Rice (accessed on 7 June 2024), Soybean (accessed on 12 June 2024), Rose (accessed on 12 June 2024) and Arabidopsis (accessed on 1 May 2024) genomes were downloaded from NCBI. Genome collinearity analysis was performed between *C. sativa* and these species using MCScanX and visualised using Dual synteny plot in Tbtools (Chen et al., 2020).

### 2.7 Identification of putative herbicide-metabolizing cytochrome P450s in *C. sativa* and herbicide selection

A literature survey was carried out to identify the articles containing the sequences of herbicide metabolizing cytochrome p450s in different plant species. Cytochrome p450 sequences and ALS inhibitor herbicides from all the papers were compiled. BLASTP search was carried out by using the protein sequences from the above cytochrome p450s to identify the closest homologs in *C. sativa*. Representatives from each chemical class of ALS-inhibiting herbicides were chosen for further analysis. 3D structure of these herbicides was downloaded from Pubchem website (accessed on 20 May 2024) and converted to pdb format using Pymol.

### 2.8 Homology modeling and molecular docking of CsCYPs

The identified CsCYP proteins were modeled using a protocol from a plant P450 database (Wang et al., 2021). Amino acid sequences in FASTA format were input into a web-based service utilizing PCPCM to generate models (accessed on 14 June 2024), which included the addition of heme groups. The structural quality of these models was then evaluated using Swiss-model’s Structure Assessment Tools (accessed on 20 June 2024). Molecular docking of cytochrome p450 with the herbicides was performed using AutoDock Tools (version 1.5.6) (Abdollahi et al., 2021). For each herbicide conformer, the Lamarckian genetic algorithm was performed with 100 independent runs and 300 population size. The process was repeated for all the CsCYPs with different herbicides. Finally, the docking results were visualized using the Discovery Studio Visualizer v21.1 and Pymol. The heme distances were measured between the closest atom of herbicide and the heme iron as these atoms are most likely to be attacked by heme iron due to close proximity.

## 3 Results

### 3.1 Identification and characterisation of CsCYPs

**Figure 1:**
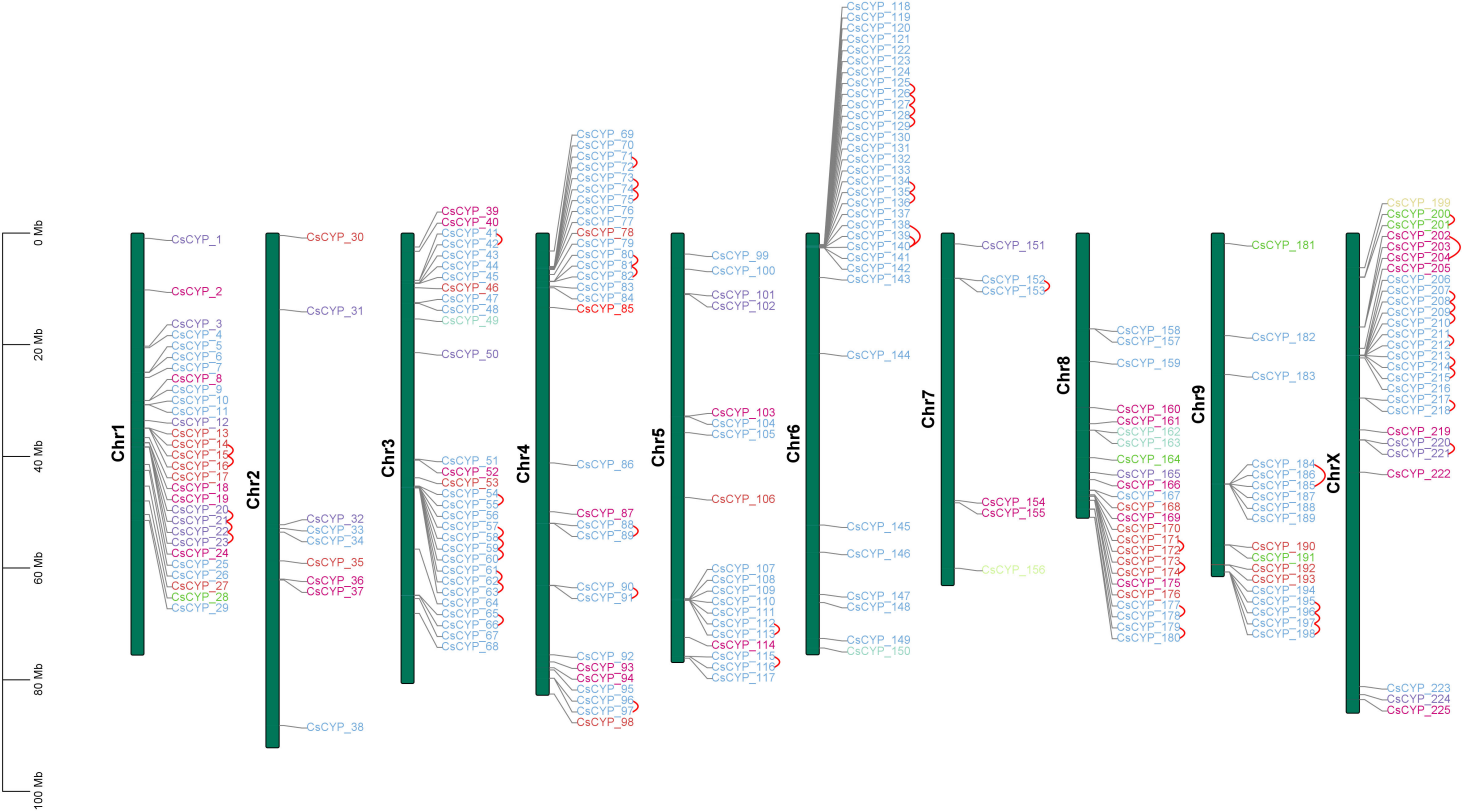
Chromosomal distribution of *C. sativa* CYPs across 10 chromosomes. Tandem duplications are indicated by arcs. Each CsCYP is color-coded according to its clan, consistent with the scheme used in **Figure 2**.

HMMER identified 239 protein sequences as cytochrome p450s using PF00067.hmm domain file and Arabidopsis cytochrome p450 sequences. BLASTP also resulted in 239 sequences. Out of these, 11 sequences were removed manually after performing alignment as they were 100% identical to the already existing sequences. 3 more sequences were removed as they had 100% identity but varied in sequence length. The sequences with the smallest length i.e., having the most common amino acids between the two were kept for further analysis to reduce redundancy. After the screening, a total of 225 cytochrome p450 protein sequences were identified which were encoded by 221 genes due to alternate splicing events. Proteins were named from 1 to 225 according to their position on chromosomes (**Figure 1**).

### 3.2 Physicochemical properties and subcellular localisation prediction and Chromosomal location analysis

An analysis of the physicochemical properties of the 225 CsCYP proteins revealed significant diversity. The proteins varied in length from 81 to 611 amino acids, with molecular weights spanning 9.49 kDa (CsCYP_36) to 69.29 kDa (CsCYP_162). Their theoretical isoelectric points ranged from slightly acidic 5.07 (CsCYP_56) to moderately basic 9.61 (CsCYP_224), with over 78% of the proteins having a basic nature (isoelectric point > 7). The stability of these proteins, as indicated by their instability index, varied considerably. Values ranged from 57.04 (CsCYP_211) to 15.44 (CsCYP_36), with 114 proteins classified as stable (index < 40) and 111 as unstable. The aliphatic index showed variation from 76.18 (CsCYP_155) to 107.28 (CsCYP_9). Hydrophilicity levels differed among the proteins, ranging from -0.581 (CsCYP_36) to 0.166 (CsCYP_221). Based on the Grand Average of Hydrophobicity (GRAVY), only 8 out of 225 proteins were hydrophobic, while the majority exhibited hydrophilic nature. Additionally, upon analysing the physicochemical properties between clan-specific CsCYPs, there were distinct differences in the properties among the clans as given in **Supplementary Table S2**, with Clan 97 exhibiting unique characteristics such as the highest average amino acid length and molecular weight, coupled with the lowest average isoelectric point. These clan-specific differences likely reflect the diverse functional roles of CsCYPs within cellular systems. BUSCA subcellular localization predictions suggested that most CsCYP proteins (213) were associated with the endomembrane system. Other predicted locations included the chloroplast outer membrane (4), organelle membrane (4), nucleus (3), and plasma membrane (1). Transmembrane region analysis using TMHMM showed that 35 proteins lacked transmembrane helices, while the rest had at least one. Notably, all 12 proteins not predicted to be in the endomembrane system were among these 35 proteins. This diverse array of structural features highlights the varied functional roles of CsCYPs in Cannabis growth and development. Cytochrome p450 genes were present on all chromosomes of which chromosomes 2 and 7 had least number of genes i.e., 9 and 6 genes respectively.

### 3.3 Phylogenetic analysis of the CsCYPs

The unrooted phylogenetic tree was constructed using the Maximum Likelihood method with 5000 ultrafast bootstraps to analyse the evolutionary relationships and classifications of putative CsCYPs. From the analysis, CsCYPs were classified into 9 clans and 47 families which can be further classified into two clades: A-type containing Clan 71 and rest all 8 clans to Non A-type clade (**Supplementary Table S3**). However, eight families- 6 from *Arabidopsis thaliana* (CYP702, CYP708, CYP709, CYP705, CYP712, CYP83) and 2 from Oryza sativa (CYP727, CYP729) were not found in *C. sativa*.

**Figure 2:**
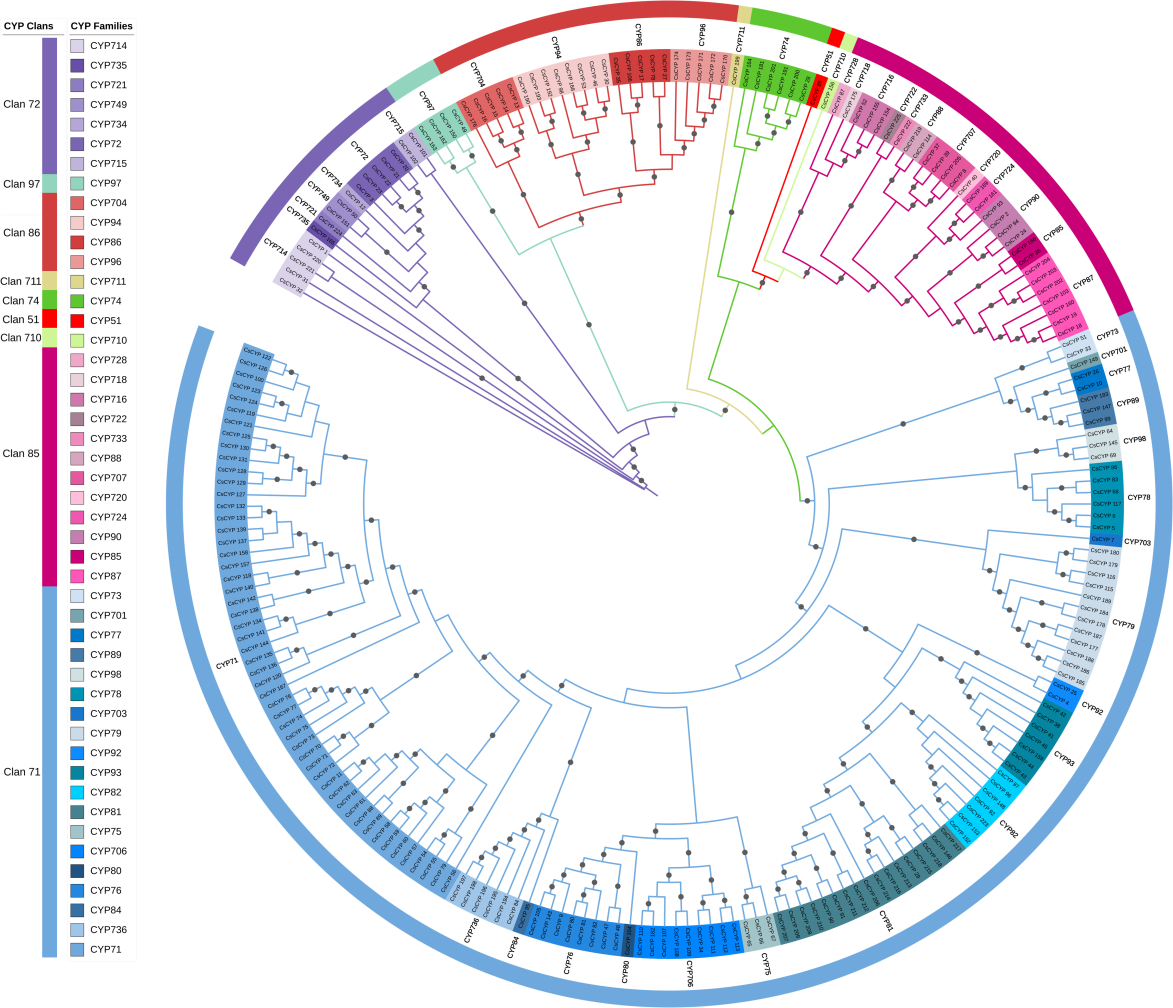
Phylogenetic analysis of CsCYP genes in *C. sativa*. A maximum likelihood tree was constructed using CsCYP protein sequences with 5000 bootstrap replicates. Gray circles at branch points indicate bootstrap values exceeding 0.7. Clans and families are delineated by color strips and symbols, respectively.

A phylogenetic analysis of *C. sativa* cytochrome P450 proteins (CsCYPs) revealed a complex evolutionary relationship among the various clans (**Figure 2**). The analysis showed that clans 86 and 97 clustered together, while clans 51, 85, 710, and 74 formed a separate clade. Interestingly, clans 711 and 71 constituted a distinct single-clan cluster. The study highlighted significant diversity within certain clans, particularly clan 71, which encompassed 19 families and 143 genes, making it the most diverse. Other multi-family clans included clan 72 (7 families, 17 genes), clan 85 (12 families, 29 genes), and clan 86 (4 families, 23 genes). In contrast, clans 51, 74, 97, 710, and 711 each comprised a single family and up to six genes per clan. This phylogenetic analysis provides valuable insights into the evolutionary dynamics and functional diversity of CsCYPs in Cannabis, suggesting potential specialization and expansion of certain clans in response to environmental or developmental pressures.

### 3.4 Gene structure and conserved motif identification

**Figure 3:**
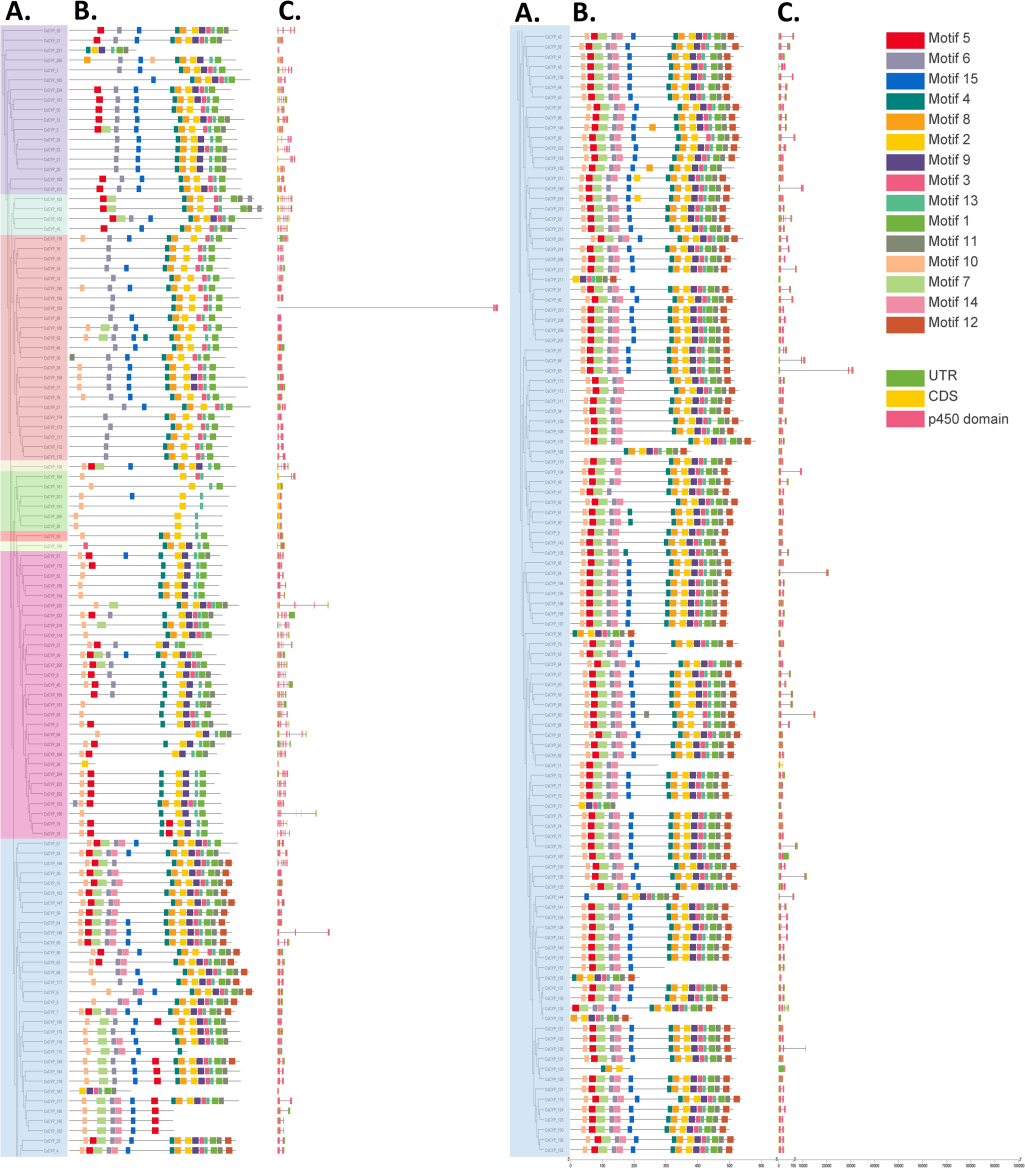
Comprehensive analysis of *C. sativa CYP* genes. **(A)** Maximum likelihood phylogenetic tree of CsCYP proteins constructed using full-length sequences (5000 bootstrap replicates). **(B)** Distribution of 15 conserved motifs in CsCYP proteins. **(C)** Gene structure of CsCYPs, depicting introns (black lines), exons (yellow rectangles), P450 domains (pink rectangles), and untranslated regions (UTRs, green rectangles).

**Figure 4:**
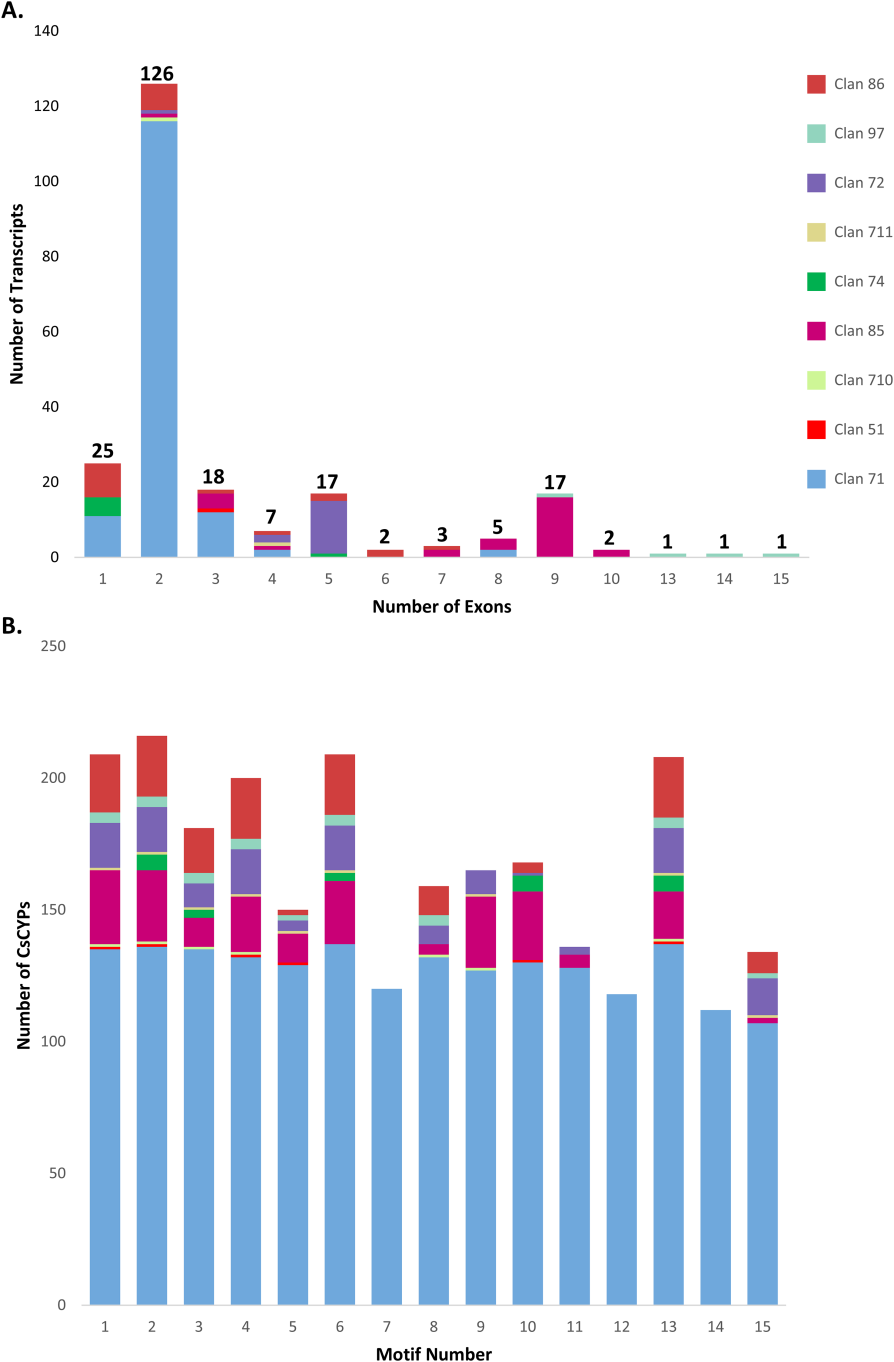
Structural analysis of *C. sativa* CYP clans. **(A)** Exon distribution patterns within CYP transcripts across nine clans. **(B)** Distribution of 15 conserved motifs across nine CYP clans.

The exon-intron structure and P450 domains of CsCYPs were visualized using the “Gene structure view” tool in Tbtools-II software. This analysis revealed significant structural diversity among the CsCYPs, particularly between A-type and non-A-type CsCYPs. The CDS-UTR composition showed high variability in non-A-type CsCYPs compared to A-type CsCYPs, while gene structures within identical CYP families exhibited similarities (**Figure 3**). A-type CYPs displayed a range of one to eight exons, with 81% (116 out of 143) containing two exons. In contrast, non-A-type CYPs showed greater variability, with exon numbers ranging from one to fifteen. For instance, clan 85 possessed two to ten exons, clan 86 had one to seven exons, and clan 97 contained nine to 15 exons. The overall distribution of exon numbers in CsCYPs was as follows: 25 CYPs with single exons, 126 CYPs (approximately half of the total) with two exons, 19 CYPs with three exons, 50 CYPs with four to nine exons, and 5 CYPs with ten to 15 exons (**Figure 4**, **Supplementary Table S1**).

The MEME online tool was employed to predict fifteen conserved motifs in CsCYPs, as illustrated in **Figure 3** and detailed in **Supplementary Table S4**. All fifteen motifs were identified in A-type CYPs, while non-A-type CYPs exhibited between 6 and 12 motifs. Notably, Motif7, Motif12, and Motif14 were found exclusively in Clan 71 (**Figure 4**, **Supplementary Table S5**). The analysis revealed various structural attributes of the CYPs’ conserved domains. The heme-binding domain, crucial for the core catalytic center, was predominantly located within Motif1, present in 92.89% of CsCYPs. The I-helix, involved in oxygen binding and activation, was primarily found within Motif4, occurring in 88.89% of CsCYPs. Motif2 contained the K-helix in 96% of CsCYPs, while the PERF motif was identified within Motif13 in 92.44% of CsCYPs; together, these form the ERR triad, thought to stabilize the highly conserved three-dimensional structure. Motif10, present in 74.67% of CsCYPs, featured a proline-rich region considered to act as a membrane hinge for correct orientation. Motif6 included a C-helix region where W and R residues interact with a propionate side chain of the heme. The remaining motifs exhibited varying degrees of conservation across the CsCYPs. This comprehensive motif analysis suggests that CsCYPs sharing the same motifs may be associated with similar biological functions, providing valuable insights into the structural and functional diversity of these proteins in *C. sativa*.

This analysis highlighted substantial variation in conserved motif patterns and gene structures between A-type and non-A-type CYPs. However, similar patterns were observed within clans or families, lending support to the phylogenetic relationships and group classifications established in this study.

### 3.5 Analysis of CsP450s cis-acting regulatory elements

**Figure 5:**
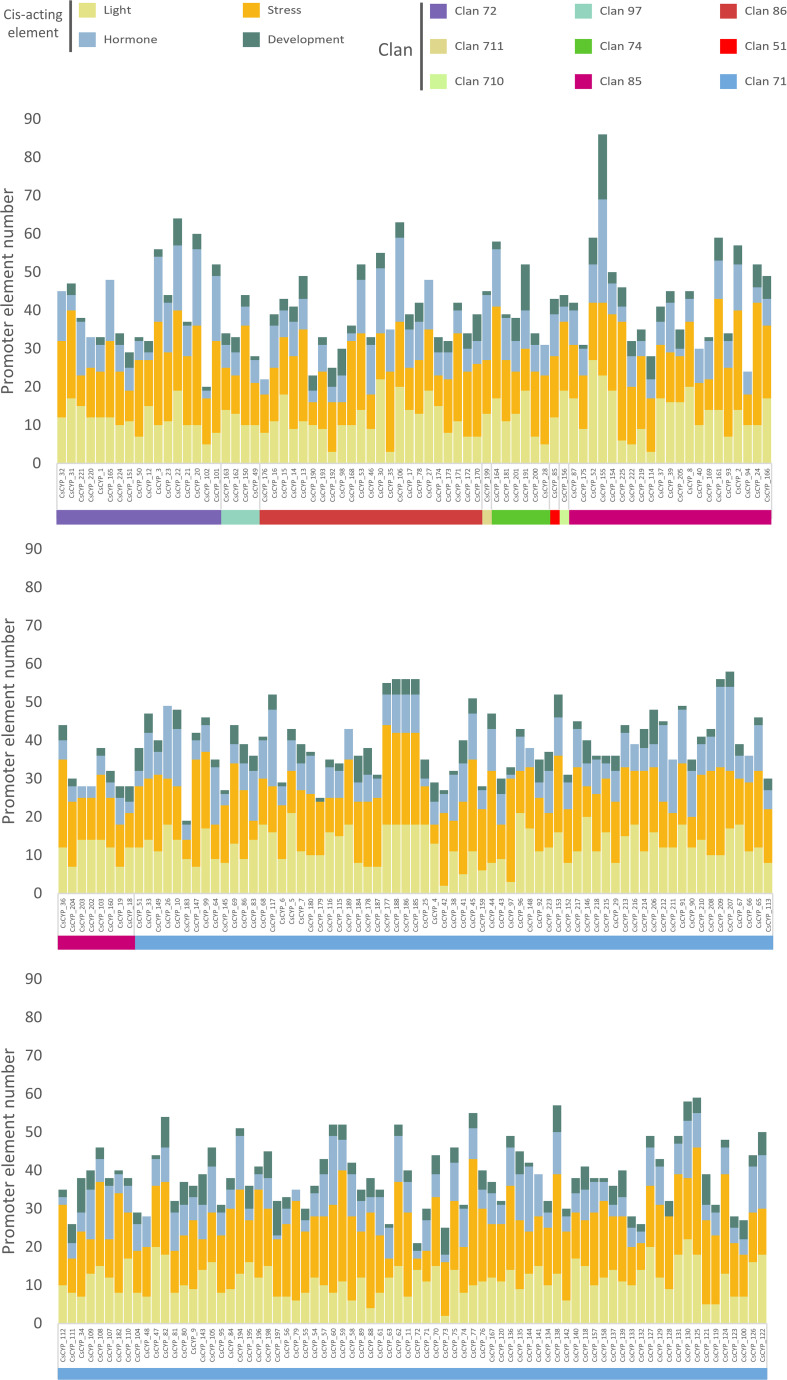
Distribution of cis-acting regulatory elements in the promoter regions of *C. sativa* cytochrome P450 genes across nine clans. Predicted cis-acting regulatory elements were categorized into four different groups: developmental, stress-responsive, hormone-responsive, and light-responsive elements.

The promoter regions of the *C. sativa* cytochrome P450 gene family were analyzed using TBtools by extracting 2000 bp upstream sequences, followed by cis-acting element analysis using the PlantCARE database. This investigation revealed 115 types of promoter elements within the CsCYP gene family (**Supplementary Table S6**). These elements were categorized into four main groups based on their responsiveness: development (22 types), stress (19 types), hormone (17 types), and light responsiveness (35 types). Stress-responsive elements were the highest in number (3642), followed by light (2706), hormone (1852), and development (735) responsive elements, indicating a significant role of CsCYPs under stress conditions (**Supplementary Table S1**). No obvious differences were observed among different clans, with all CsCYPs possessing light and stress-responsive cis-acting elements. However, 2 CsCYPs lacked hormone-responsive elements, and 20 lacked development-responsive elements (**Figure 5**).

Among the 35 light-responsive elements predicted, the four most frequent were Box 4 (96.89%, 218 out of 225), G-box (73.33%), GT1-motif (59.11%), and TCT-motif (54.22%). Hormone-responsive elements were also identified, with the ethylene-responsive element (ERE) being the most common (180 out of 225), followed by the abscisic acid (ABA)-responsive element (ABRE) (153). Other hormone-responsive elements included those for auxin (TGA-Box, TGA-element, and AuxRR-core), methyl jasmonate (MeJA) (TGACG-motif and CGTCA-motif), gibberellin (P-box, GARE-motif, and TATC-box), and salicylic acid (TCA-element and SARE). Stress-responsive elements related to anaerobic conditions (ARE), drought (DRE1, DRE core, and MBS), wounding (box S, WRE3, W box, and WUN-motif), and low-temperature (LTR) were found in 76.89% (173), 47.56% (107), 89.33% (201), and 34.22% (77) of CYPs, respectively. Development-responsive elements such as the AAGAA-motif, O2-site, CAT-box, circadian, and GCN4_motif, associated with endosperm-specific negative expression, zein metabolism regulation, meristem expression, circadian control, and endosperm expression, were identified in 66.22% (149), 32.44% (73), 30.67% (69), 17.33% (39), and 16.89% (38) of CsCYPs, respectively (**Supplementary Table S1**).

The abundance and diversity of these cis-acting elements suggest that they may play crucial roles in regulating CsCYP expression during development and in response to various environmental stimuli such as light, stress, and hormones. This comprehensive analysis provides insights into the potential involvement of CsCYPs in diverse biological processes and regulatory pathways in *C. sativa*, highlighting their adaptability and functional significance in plant growth, development, and stress responses.

### 3.6 Collinearity and Synteny analysis

**Figure 6:**
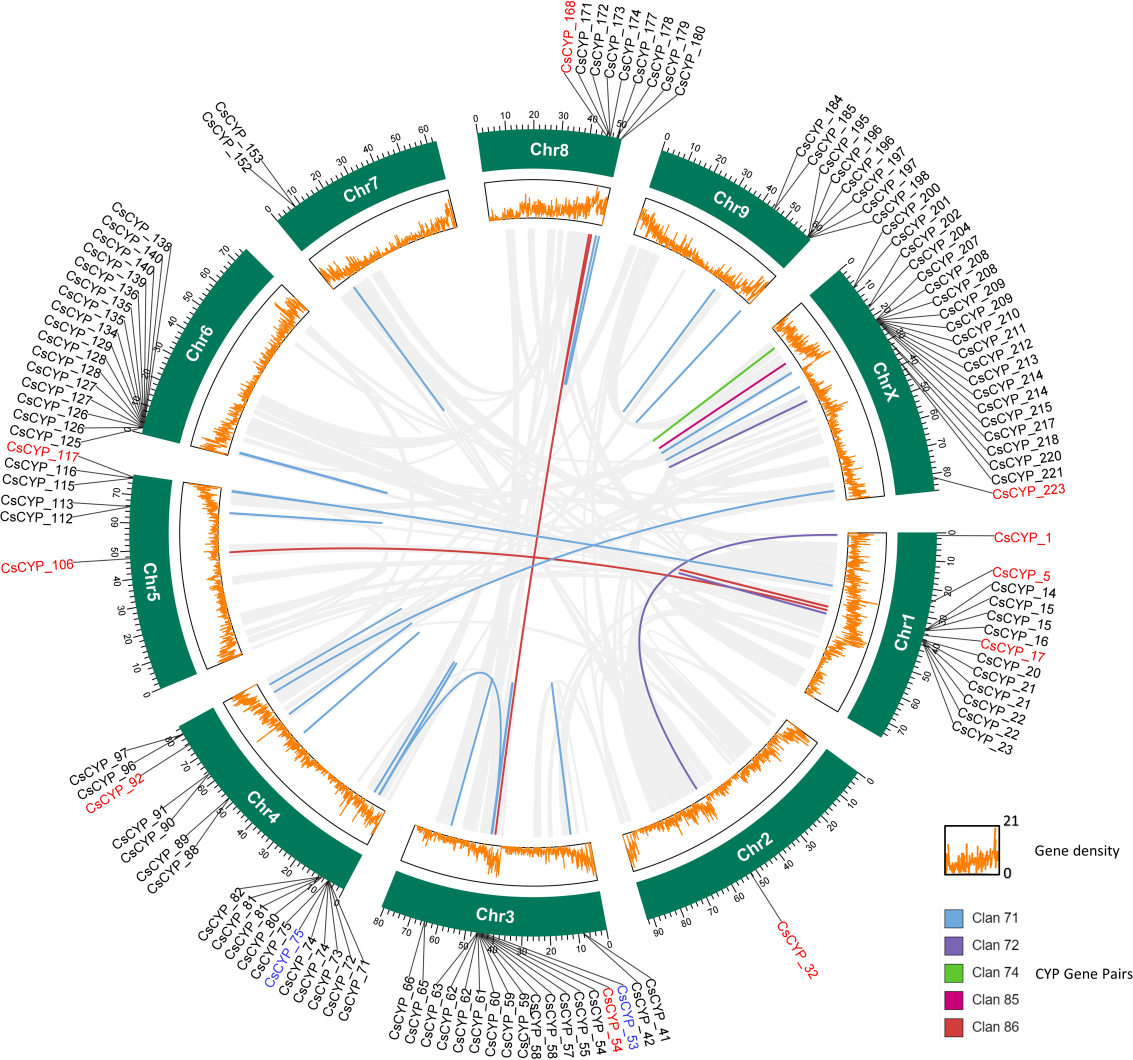
Visualization of clan-specific gene duplication events among *C. sativa* CYP genes. Duplicated gene pairs are depicted in a circos diagram, with clan-specific color coding corresponding to **Figure 2**. Segmental duplications (red labels) span different chromosomes, while tandem duplications (black labels) occur within the same chromosome. Genes undergoing both segmental and tandem duplication are labeled in blue. Light gray lines represent syntenic blocks in the *C. sativa* genome. The outer track (green) denotes chromosomes, while the inner track (orange) illustrates gene density.

The evolution of genomes and genes is known to be significantly influenced by gene duplication. In order to better understand the evolution and spread of the P450 gene families in the Cannabis plant, the investigation of gene duplication is essential. Thus, collinearity and gene duplication in the Cannabis genome were analysed using MCScanX. The duplication gene analysis revealed 50 pairs of CsCYPs were tandemly duplicated and 6 pairs of CsCYPs were collinear, signifying their pivotal role in the expansion of the CsCYP gene family (**Figure 6**, **Supplementary Table S7**). Moreover, 36 and 88 CsCYPs duplicated from proximal and dispersed events, respectively (**Supplementary Table S8**).

**Figure 7:**
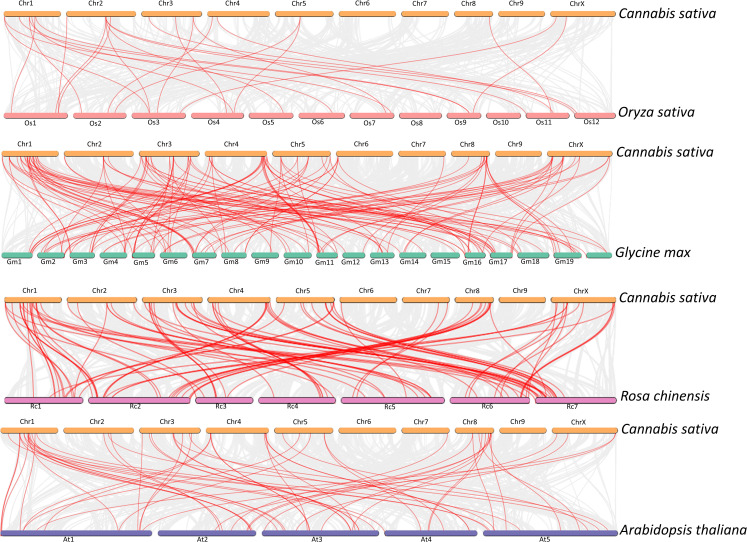
Collinearity analysis of CsCYPs in *C. sativa* compared to other plant species: rice, soybean, China rose and Arabidopsis. Red lines indicate homologous CYP450 gene pairs between genomes, while gray lines represent collinear genomic blocks.

To further investigate the origin and evolutionary relationship of CsCYP genes, collinearity analysis was performed between other species (**Figure 7**). Of the 225 CsCYPs, 19 were collinear with rice genes, 71 with soybean genes, 78 with rose genes and 44 with Arabidopsis genes. Number of collinear species match the relationship on the phylogenetic distribution of their orders on the phylogeny, thus the origin must have happened before the divergence of these plants but the expansion happened with the evolution of these plants.

### 3.7 Identified putative herbicide metabolizing CsCYPs

After the extensive literature survey, 22 cytochrome p450s were identified from different research papers which have been shown to metabolize 18 different ALS herbicides (**Supplementary Table S9**). Blastp of these cytochrome p450s resulted in 15 CsCYPs of varying lengths. CsCYPs having protein length greater than 500 amino acid residues were kept for further analysis resulting in 14 CsCYPs, thus excluding CsCYP_144 (356 a.a.) and CsCYP_211 (160 a.a.). Herbicides were further shortlisted to represent 2 molecules from each of the 6 classes of the ALS herbicides resulting in 9 molecules (**Supplementary Table S10**).

### 3.8 Homology modeling and molecular docking

In order to study the interactions between the herbicides and Cannabis cytochrome p450s, thirteen CsCYPs were selected for homology modeling and docking studies. Final structures of these CsCYPs had a QMEANDisCo score between 0.59 and 0.68 (**Supplementary Table S11**), comparable with valuation of previously reported cytochrome p450 models (Abdollahi et al., 2021; Chen et al., 2023). Further structure assessment showed 94.79% to 98.41% Ramachandran favoured residues and outliers ranged from 0% to 1.4%. According to previous studies, models having greater than 90% residues in the favoured region of the plot are considered to be good quality protein models (Pramanik et al., 2017; Rani and Pooja, 2018; Chen et al., 2023).

**Figure 8:**
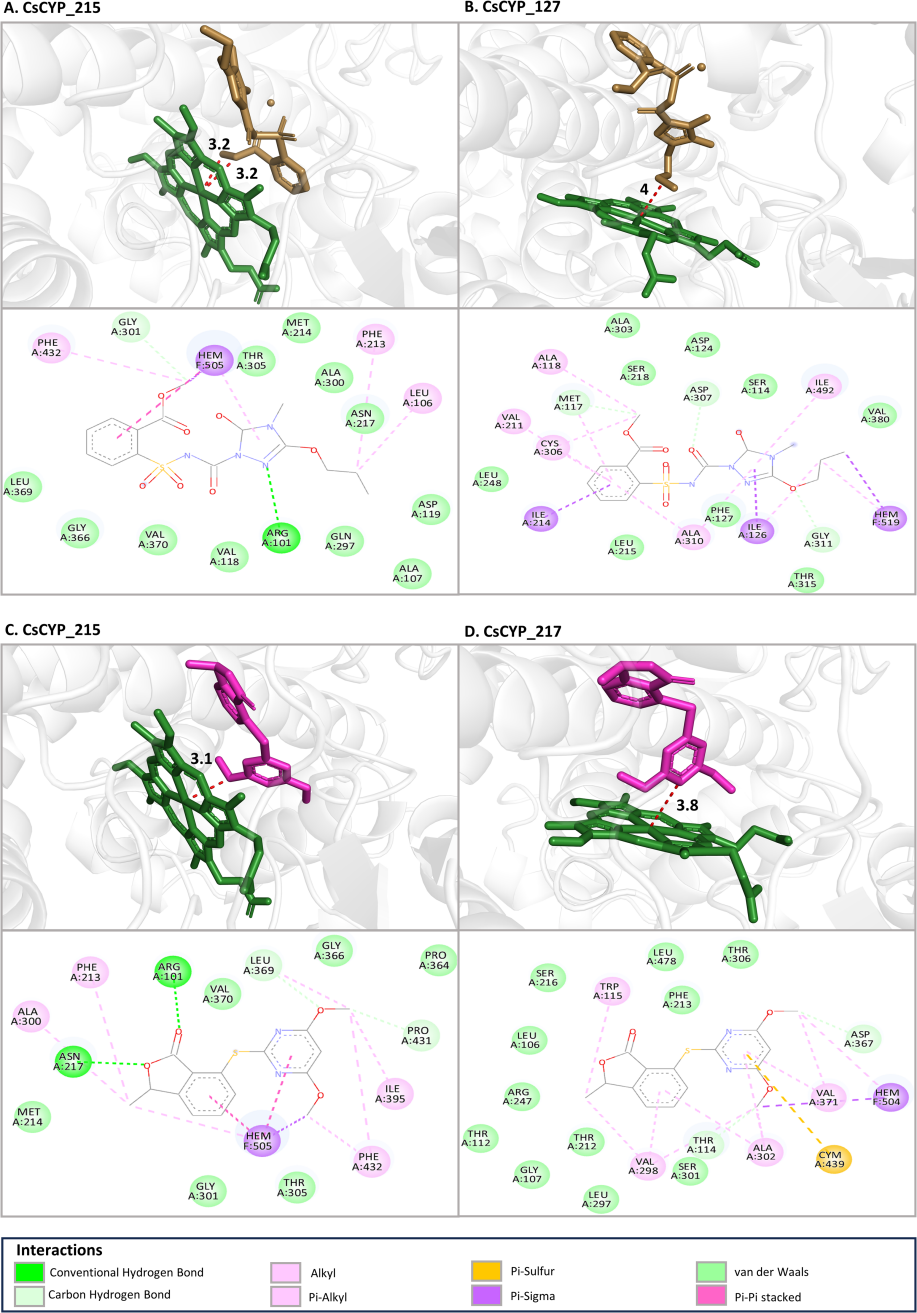
The binding mode of top four lowest binding affinity docking with heme distance less than 6Å **(A)** CsCYP_215 with Propoxycarbazone-sodium (-9.31 kcal/mol), **(B)** CsCYP_127 with Propoxycarbazone-sodium (-7.88 kcal/mol), **(C)** CsCYP_215 with Pyriftalid (-7.88 kcal/mol) and **(D)** CsCYP_217 with Pyriftalid (-7.84 kcal/mol) at the active site of CsCYPs. For each docking with a herbicide, the top image shows the 3D view of shortest distance between the herbicide and the iron in heme group and the bottom image shows the interacting residues in 2D.

The study also investigated the binding affinities and interaction patterns between modelled CsCYPs and different ALS inhibiting herbicides through molecular docking simulations. Among the herbicides tested, propoxycarbazone-sodium, pyriftalid (**Figure 8**), and bispyribac-sodium consistently demonstrated strong binding energies with multiple CsCYPs. These herbicides exhibited binding energies below -6 kcal/mol with at least ten different CsCYP proteins, indicating their potential as substrates for these enzymes (**Table 1**). In contrast, penoxsulam, pyrimisulfan and pyroxsulam consistently showed lower negative binding energies with all CsCYPs except CsCYP_217. Among the CsCYPs, CsCYP_215, CsCYP_217, CsCYP_213 and CsCYP_14 had the highest average binding energies of -6.933 kcal/mol, -6.922 kcal/mol, -6.735 kcal/mol and -6.677 kcal/mol respectively, among the herbicides. The heme distances observed in the docking results varied considerably across different CsCYP-herbicide combinations, ranging from as close as 2.5Å to as far as 13.9Å with 55 docking results having heme at a distance less than 6Å and 19 results with heme distance of greater than 6Å, predominantly in CsCYP_128 and CsCYP_26 (**Table 2**).

**Table 1:** Binding energies in kcal/mol of various docking results with different herbicides and proteins.

Proteins	Bispyribac-sodium (kcal/mol)	Bensulfuron-methyl (kcal/mol)	Imazamox (kcal/mol)	Mesosufuron-methyl (kcal/mol)	Propoxycarbazone-sodium (kcal/mol)	Pyriftalid (kcal/mol)	Penoxsulam (kcal/mol)	Pyrimisulfan (kcal/mol)	Pyroxsulam (kcal/mol)
CsCYP_14	-6.83	-6.42	-7.48	-5.81	-6.75	-6.77	-5.03	-5.83	-5.64
CsCYP_20	-5.94	-5.19	-6.68	-5.75	-7.04	-6.1	-5.24	-4.21	-4.15
CsCYP_21	-6.06	-5.85	-6.5	-5.74	-6.82	-6.68	-4.74	-4.67	-3.66
CsCYP_23	-6.22	-6.03	-6.42	-6.74	-6.75	-6.53	-4.54	-4.93	-5.04
CsCYP_26	-6.37	-6.62	-5.88	-6.28	-7.19	-6.53	-4.99	-4.94	-5.79
CsCYP_50	-6.54	-5.42	-6.74	-5.34	-7.05	-6.55	-3.92	-4.7	-4.64
CsCYP_127	-6.06	-6.6	-5.21	-6.26	-7.88	-6.44	-5.95	-4.43	-5.99
CsCYP_128	-6.5	-5.69	-5.95	-6.35	-6.49	-6.46	-4.67	-4.56	-3.77
CsCYP_210	-5.75	-6.35	-5.4	-5.78	-6.4	-6.59	-5	-3.91	-5.79
CsCYP_213	-7.25	-6.01	-6.21	-6.95	-7.22	-6.77	-5.45	-4.63	-5.47
CsCYP_215	-6.42	-6.4	-6	-5.59	-9.31	-7.88	-5.52	-4.89	-5.51
CsCYP_217	-6.59	-5.68	-6.14	-8.1	-7.18	-7.84	-6.35	-5.7	-6.32
CsCYP_218	-5.87	-6.36	-5.77	-6.43	-6.95	-6.98	-4.29	-5.68	-5.02

The gradient in the table represents binding energies, with darker shades of green indicating stronger binding (more negative binding energy values), and lighter shades representing weaker binding (less negative binding energy values).

**Table 2:** Distance of heme in angstrom from various herbicides in different proteins and common interacting residues in three or more different binding modes.

Proteins/herbicides	Bispyribac-sodium (Å)	Bensulfuron-methyl (Å)	Imazamox (Å)	Mesosufuron-methyl (Å)	Propoxycarbazone-sodium (Å)	Pyriftalid (Å)	Common interacting residues in 3 or more binding modes
CsCYP_14	5.2	4.7	5.9	6.6	4.7	4.7	Asn100, Asp373, Gly374, Heme, Ile295, Ile484, Leu104, Leu108, Leu394, Lys96, Phe113, Tyr216, Thr300, Val370, Val372, Val396
CsCYP_20	6	2.8	3	7.6	3.3	3.2	Ala139, Ala143, Ala334, Arg403, Heme, Ile509, Leu330, Leu400, Leu401, Lys128, Phe333, Thr338, Thr507, Val398, Val402,
CsCYP_21	3.2	2.7	2.7	3.5	3.1	3.7	Ala143, Ala334, Asn144, Cym472, Heme, Ile135, Ile509, Leu138, Leu330, Leu401, Lys128, Phe333, Ser400, Thr338, Thr507, Val139, Val398
CsCYP_23	3.7	2.7	3.4	2.5	3.9	3.8	Ala337, Ala401, Heme, Ile402, Ile513, Leu333, Leu512, Lys131, Phe336, Phe403, Pro400, Ser146, Thr341, Thr510
CsCYP_26	11.5	3.5	3.5	11.6	13.6	8.2	Arg110, His55, His374, Leu217, Leu377, Phe375, Phe489, Phe491, Pro104, Pro372, Ser373, Ser378, Thr492, Val493
CsCYP_50	4.4	3.8	3.4	5.5	2.5	2.7	Ala133, Ala324, Asn498, Heme, Ile141, Ile500, Leu389, Leu391, Lys130, Met134, Met147, Met239, Phe323, Ser390, Thr328, Thr392, Thr501, Val137, Val146, Val388
CsCYP_127	6.6	2.7	2.3	3.5	4	3.3	Ala310, Ala376, Gly311, Gly491, Heme, Ile126, Ile214, Ile492, Leu215, Leu378, Leu379, Phe127, Phe490, Pro377, Pro381, Ser218, Thr315, Val380
CsCYP_128	9.6	6.5	10	6.4	8	10.4	Ala217, Ala306, Ala311, Ala376, Ala377, Arg108, Asn111, Asp223, Gly310, Gly492, Ile125, Ile493, Leu379, Leu402, Lys400, Met378, Phe126, Phe380, Phe491, Pro381, Ser216, Ser307, Val113, Val213
CsCYP_210	5.3	2.9	2.6	5.7	10.4	3.6	Ala304, Ala308, Arg102, Gly108, Gly112, Gly227, Heme, Ile111, Ile119, Ile223, Leu307, Leu377, Met481, Ser107, Ser120, Thr117, Thr312, Val303
CsCYP_213	7.8	4.1	12	2.9	3.1	3	Ala225, Ala313, Ala378, Arg103, Gly224, Gly491, Heme, His57, Leu52, Leu312, Leu380, Leu381, Leu492, Phe75, Pro379, Pro383, Thr317, Thr493, Val56, Val382
CsCYP_215	2.5	5.2	4.9	13.8	3.2	3.1	Arg101, Asn217, Gly301, Gly366, Heme, Leu369, Leu392, Leu478, Met214, Phe104, Phe432, Pro371, Thr305, Val370
CsCYP_217	2.8	4.9	2.7	12.5	13.8	3.8	Ala302, Arg247, Asp367, Heme, Leu106, Leu297, Leu478, Phe213, Ser216, Ser301, Thr114, Thr212, Thr306, Trp115, Val298, Val371
CsCYP_218	6.1	4	3.6	12	4.5	3.3	Ala102, Ala308, Ala312, Arg248, Gly217, Gly377, Heme, Leu106, Leu214, Leu307, Leu380, Leu489, Ser311, Thr213, Thr316, Trp115, Val114, Val376

Distances up to 6 Å are shaded with the same green color, indicating that they fall within an acceptable interaction range. Values above 6 Å are displayed in a gradient of lighter shades, with lighter colors representing increasing distances.

Furthermore, analysis of the interacting residues across these enzyme-herbicide complexes revealed some common features. Common residues frequently involved in interactions across multiple herbicides included Ala, Arg, Asn, Asp, Gly, Ile, Leu, Lys, Met, Phe, Pro, Ser, Thr and Val (**Table 2**) whereas Cys, Glu, Gln, His, Tyr and Trp were less commonly found (**Supplementary Table S12**). The heme group of the CsCYP enzymes was also often implicated in herbicide binding. Hydrophobic amino acids, particularly alanine, leucine, isoleucine, phenylalanine and valine, were frequently involved in interactions such as alkyl and pi-alkyl interactions. This pattern suggests that hydrophobic interactions play a crucial role in stabilizing the herbicide-enzyme complexes across various CsCYP proteins. Aromatic residues, particularly phenylalanine, were consistently present in the binding sites of many CsCYP-herbicide complexes, indicating the importance of π-π stacking and other aromatic interactions in stabilizing the herbicide-enzyme complexes. Polar and charged residues also contributed significantly to the binding interactions. Residues such as serine, threonine, arginine, asparagine, and lysine were also involved in the formation of hydrogen bonds or electrostatic interactions with the herbicides. The consistent involvement of glycine residues in many binding sites, such as Gly374, Gly311, Gly112, Gly366, Gly217 in the CsCYP_14, CsCYP_127, CsCYP_210, CsCYP_215 and CsCYP_218 interactions respectively with multiple herbicides, suggests that conformational flexibility in certain regions of the binding pocket may be important for accommodating different herbicide structures.

## 4 Discussion

This comprehensive study provides novel insights into the cytochrome P450 (CYP) gene family in *C. sativa* and its potential role in herbicide metabolism. Through a systematic bioinformatics approach, we identified and characterized 225 CYP proteins encoded by 221 genes in the Cannabis genome. This represents one of the most extensive analyses of the CYP family in this economically important yet understudied crop species to date.

The 225 CYP proteins identified in *C. sativa* demonstrate considerable diversity in their physicochemical properties, subcellular localization, and gene structure. This diversity likely reflects the functional versatility of CYPs in plant metabolism. The majority of CsCYPs were predicted to localize to the endomembrane system, consistent with the known association of plant CYPs with the endoplasmic reticulum (Szczesna-Skorupa et al., 1998; Williams et al., 2000; Neve and Ingelman-Sundberg, 2010). However, a small number were predicted to localize to other cellular compartments (Schuler et al., 2006), suggesting potential specialized functions. Phylogenetic analysis revealed that the CsCYPs could be classified into 9 clans and 47 families. The absence of certain CYP subfamilies found in Arabidopsis and rice suggests potential lineage-specific loss or gain of CYP subfamilies during the evolution of Cannabis. This phylogenetic distribution provides a foundation for future comparative studies to elucidate the evolutionary history of plant CYPs. The gene structure analysis revealed notable differences between A-type and non-A-type CYPs, with A-type CYPs generally having a simpler exon-intron structure. This structural dichotomy has been observed in other plant species and may reflect the evolutionary history and functional divergence of these two major CYP groups (Liu et al., 2023). The conservation of gene structure within CYP families supports the reliability of our phylogenetic classification. Motif analysis identified several highly conserved regions across CsCYPs, including the heme-binding domain, I-helix, K-helix, and PERF motif. These conserved elements are crucial for the catalytic function of CYPs and their presence confirms the functional annotation of the identified sequences (Bak et al., 2011). The clan-specific distribution of certain motifs (e.g., motifs 7, 12, and 14 in Clan 71) suggests potential functional specialization among CYP clans. The analysis of cis-acting regulatory elements in CsCYP promoter regions revealed a diverse array of potential regulatory mechanisms. The abundance of stress-responsive elements suggests a significant role for CsCYPs in stress response, consistent with the known functions of CYPs in plant defence (Lee et al., 2010; Geisler et al., 2013) and stress adaptation (Mao et al., 2013; Iwakami et al., 2014; Xu et al., 2015). The presence of numerous hormone-responsive elements indicates potential involvement of CsCYPs in hormone signalling pathways, which could have implications for Cannabis growth, development, and secondary metabolism (Vadassery et al., 2008; Zhang et al., 2011). Gene duplication analysis revealed that both tandem and segmental duplication events have contributed to the expansion of the CsCYP gene family in Cannabis. The predominance of tandem duplications (50 pairs) over segmental duplications (6 pairs) suggests that local genomic rearrangements have played a major role in CYP family expansion in Cannabis. This pattern of gene family expansion through duplication is consistent with observations in other plant species (Shen and Li, 2023; Lin et al., 2024). The collinearity analysis with other plant species (Rice, Soybean, China rose, and Arabidopsis) provides insights into the evolutionary history of CsCYPs. The varying degrees of collinearity observed with these species align with their phylogenetic relationships to Cannabis, suggesting that while the origin of many CsCYPs predates the divergence of these lineages, subsequent lineage-specific expansions have occurred. This evolutionary pattern underscores the dynamic nature of CYP family evolution in plants and highlights the potential for species-specific adaptations.

A key focus of this study was to identify potential herbicide-metabolizing CYPs in Cannabis. Through homology-based identification and molecular docking simulations, we identified several CsCYPs that show promising interactions with various ALS-inhibiting herbicides. The molecular docking results revealed that certain herbicides, particularly propoxycarbazone-sodium, pyriftalid, and bispyribac-sodium, consistently demonstrated strong binding affinities with multiple CsCYPs. This suggests that these herbicides may be substrates for several Cannabis CYPs, potentially indicating a mechanism for herbicide tolerance. The binding energies observed (often below -6 kcal/mol) are comparable to those reported in other studies of CYP-herbicide interactions (Li et al., 2013; Boachon et al., 2019; Chen et al., 2023; Zou et al., 2023), supporting the biological relevance of these *in silico* predictions. The analysis of binding site residues across CsCYP-herbicide complexes revealed common features that may be important for herbicide recognition and binding. The frequent involvement of hydrophobic and aromatic residues suggests that hydrophobic interactions and π interactions play crucial roles in stabilizing herbicide-enzyme complexes, consistent with the general mechanism of CYP-mediated metabolism, where substrates often need to be positioned in a hydrophobic pocket near the heme group for oxidation to occur (Hasemann et al., 1995; Denisov et al., 2005; Gay et al., 2010; Boachon et al., 2019). Hydrogen bonding or electrostatic interactions likely play a crucial role in orienting the herbicide molecules within the active site and may contribute to the specificity of certain CsCYPs for particular herbicides. The involvement of glycine residues in many binding sites hints at the importance of conformational flexibility in accommodating diverse herbicide structures (Coleman et al., 2005; Singh et al., 2020).

According to previous studies, a substrate docked within 6Å of the heme iron is efficiently metabolised by the cytochrome p450 (de Graaf et al., 2006; Vasanthanathan et al., 2009; Huang et al., 2019). The variability in heme distances across strong interactions suggests that different CsCYPs might employ different strategies for herbicide binding and potentially for metabolism. Interestingly, the binding sites for different herbicides within the same CsCYP often showed considerable overlap, but with some variations. This suggests that these enzymes possess somewhat flexible binding pockets capable of accommodating structurally diverse herbicides consistent with the previous studies (Dimaano and Iwakami, 2021), which could contribute to their potential broad-spectrum herbicide-metabolizing capabilities.

## 5 Conclusion

This comprehensive study provides the first genome-wide characterization of the cytochrome P450 gene family in *C. sativa*, revealing 225 CYP proteins with diverse structural and functional attributes. Our integrative approach, combining phylogenetic analysis, gene structure examination, and regulatory element prediction, offers crucial insights into the evolution and potential functions of CsCYPs. Notably, molecular docking simulations and experimental validation highlight the role of CYPs in herbicide tolerance, particularly for ALS inhibitors like propoxycarbazone-sodium, pyriftalid, and bispyribac-sodium. The identification of promising herbicide-metabolizing CYP candidates such as CsCYP_14, CsCYP_213, CsCYP_215 and CsCYP_217, opens new avenues for developing herbicide-tolerant Cannabis varieties. Moreover, the abundance of stress-responsive elements in CsCYP promoters suggests their broader involvement in stress adaptation. This research not only advances our understanding of CYP-mediated herbicide metabolism in Cannabis but also provides a valuable resource for future studies on stress tolerance in this economically important crop. The findings presented here lay a solid foundation for targeted breeding and biotechnological approaches to improve Cannabis cultivation, potentially addressing key challenges in weed management and crop productivity.

## Data Availability

The original contributions presented in the study are included in the article/**Supplementary Material**. Further inquiries can be directed to the corresponding author/s.
