# Supplementary material for: Genome-wide analysis of the Cannabis sativa cytochrome P450 monooxygenase superfamily and uncovering candidate genes for improved herbicide tolerance
Source: Front Plant Sci. 2024 Nov 7;15:1490036. doi: 10.3389/fpls.2024.1490036 (PMC11578719; doi:10.3389/fpls.2024.1490036)
Supplement: Supplementary file 1 [file DataSheet1.docx]

**Genome-wide analysis of the *Cannabis sativa* cytochrome P450 monooxygenase superfamily and uncovering candidate genes for improved herbicide tolerance**

**Navneet Kaur^1^, Awadhesh Kumar Verma^1^, Madhuri Girdhar^2^, Anil Kumar^3^, Maqsood A. Siddiqui^4^, Abdulaziz A. Al-Khedhairy^4^, Tabarak Malik^5*^, Anand Mohan^1*^**

^1^School of Bioengineering and Biosciences, Lovely Professional University, Phagwara, Punjab, India

^2^Division of Research and Development, School of Bioengineering and Biosciences, Lovely Professional University, Phagwara, Punjab, India

^3^Gene Regulation Laboratory, National Institute of Immunology, New Delhi, India

^4^Chair for DNA Research, Department of Zoology, College of Science, King Saud University, P.O. Box 2455, Riyadh, Saudi Arabia.

^5^Department of Biomedical Sciences, Institute of Health, Jimma University, Ethiopia

*Corresponding E-mail: [tabarak.malik@ju.edu.et](mailto:tabarak.malik@ju.edu.et), [anandmohan77@gmail.com](mailto:anandmohan77@gmail.com)

**Supplementary information**


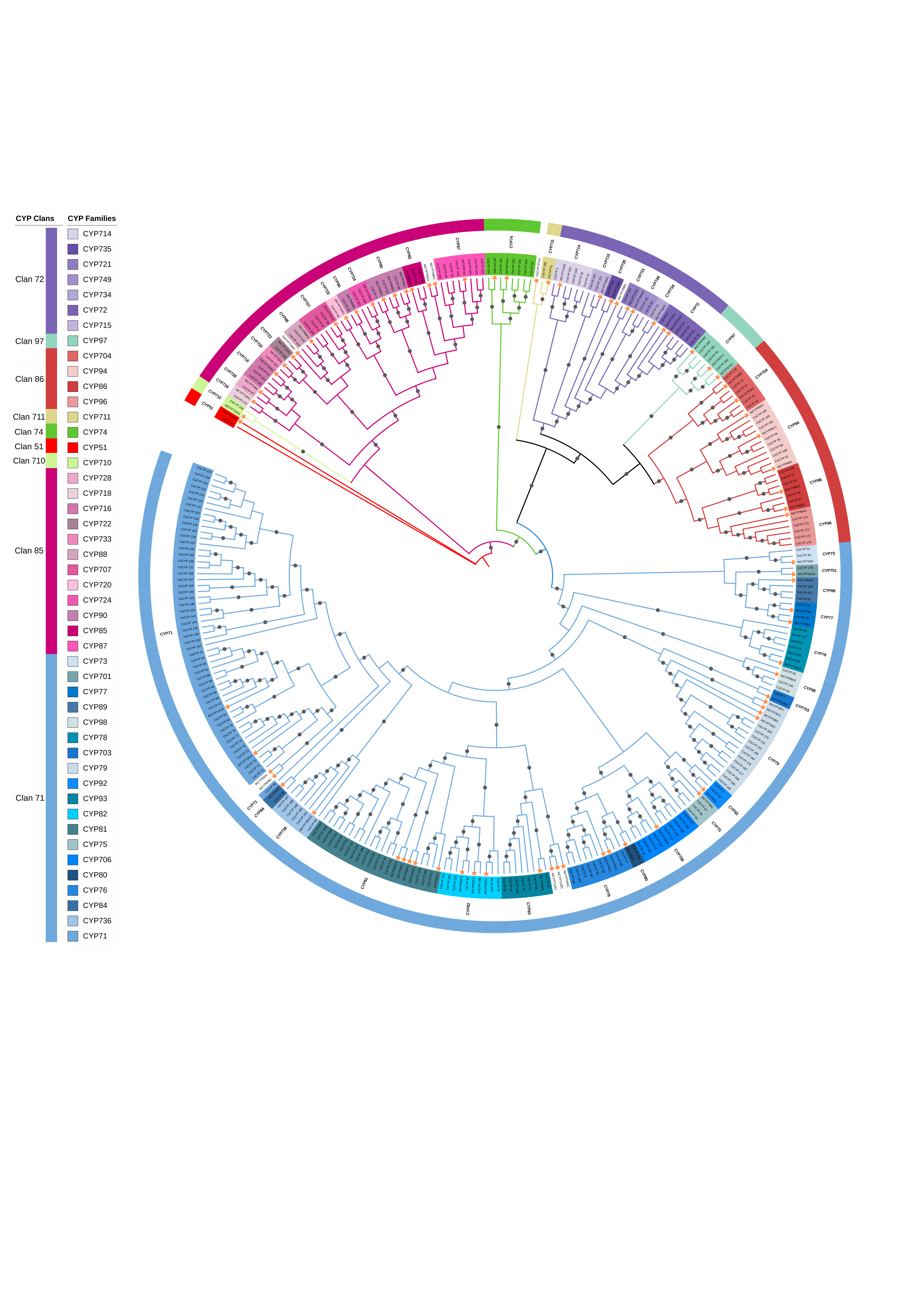


Figure S1: Phylogenetic analysis of *Cannabis sativa* CYP genes in relation to representative CYP450 genes from various species. A maximum likelihood tree was constructed using protein sequences with 5000 bootstrap replicates. Gray circles at branch points indicate bootstrap values exceeding 0.7. Clans and families are denoted by color strips and symbols, respectively. Orange stars represent CYPs from species other than *C. sativa*.


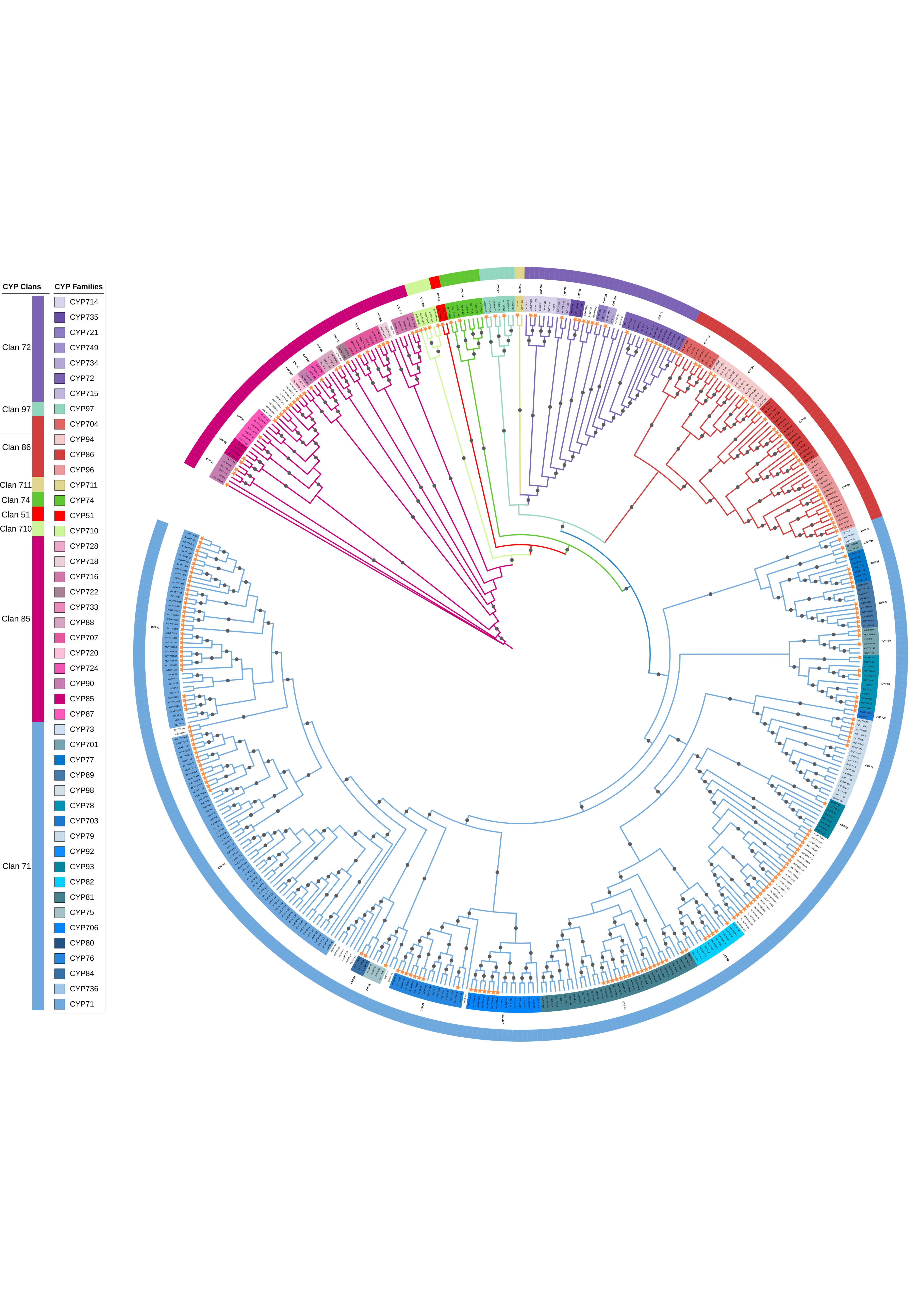


Figure S2: Phylogenetic analysis of CYP genes from *Cannabis sativa* and *Arabidopsis thaliana* (AtCYPs). A maximum likelihood tree was constructed using protein sequences with 5000 bootstrap replicates. Gray circles at branch points indicate bootstrap values exceeding 0.7. Clans and families are denoted by color strips and symbols, respectively. Orange stars represent AtCYPs.


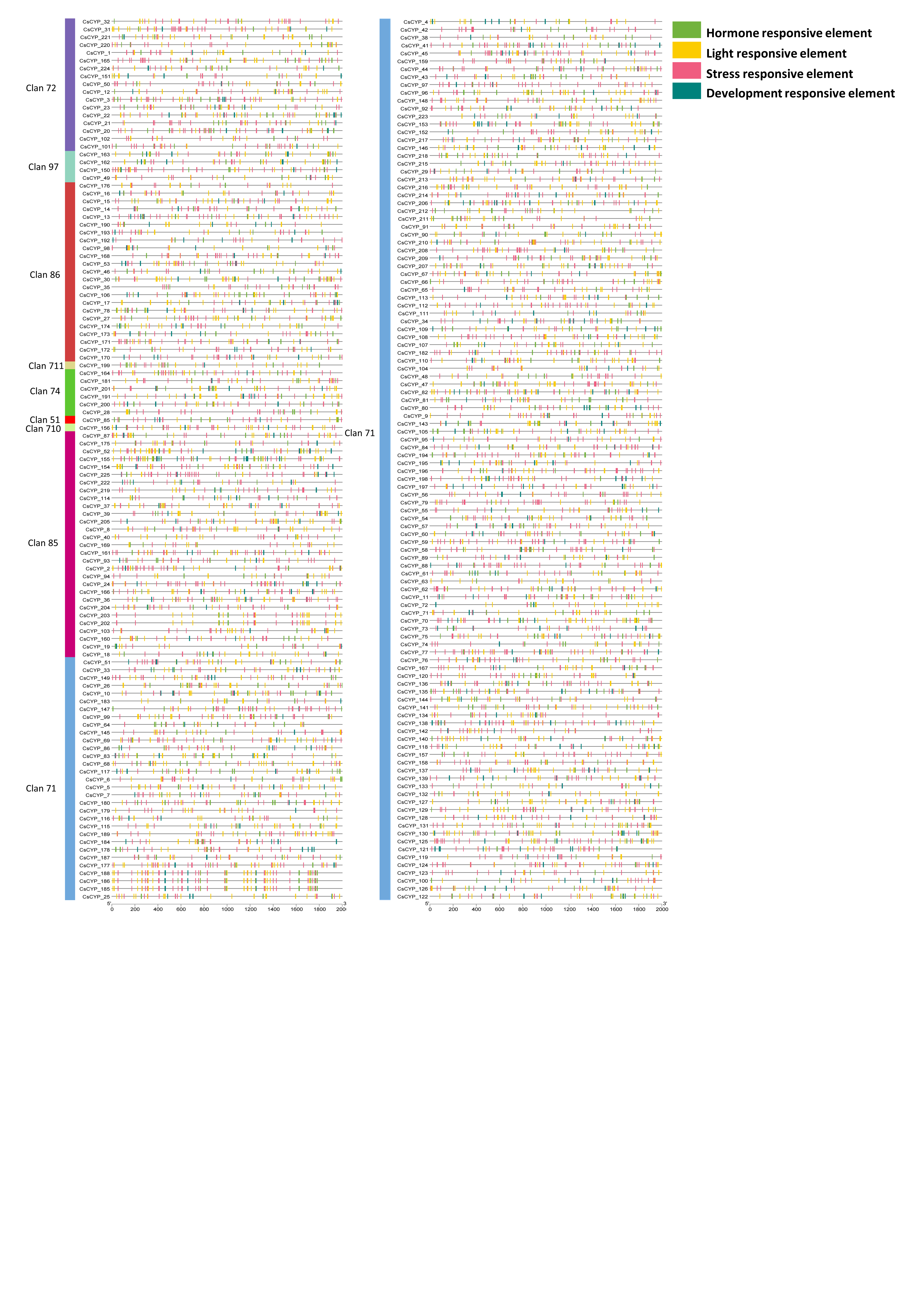


Figure S3: Analysis of cis-acting regulatory elements in the *Cannabis sativa* cytochrome p450 genes. Distribution of cis-elements identified within 2000 bp upstream promoter regions of CsCYP genes, categorized into four groups.


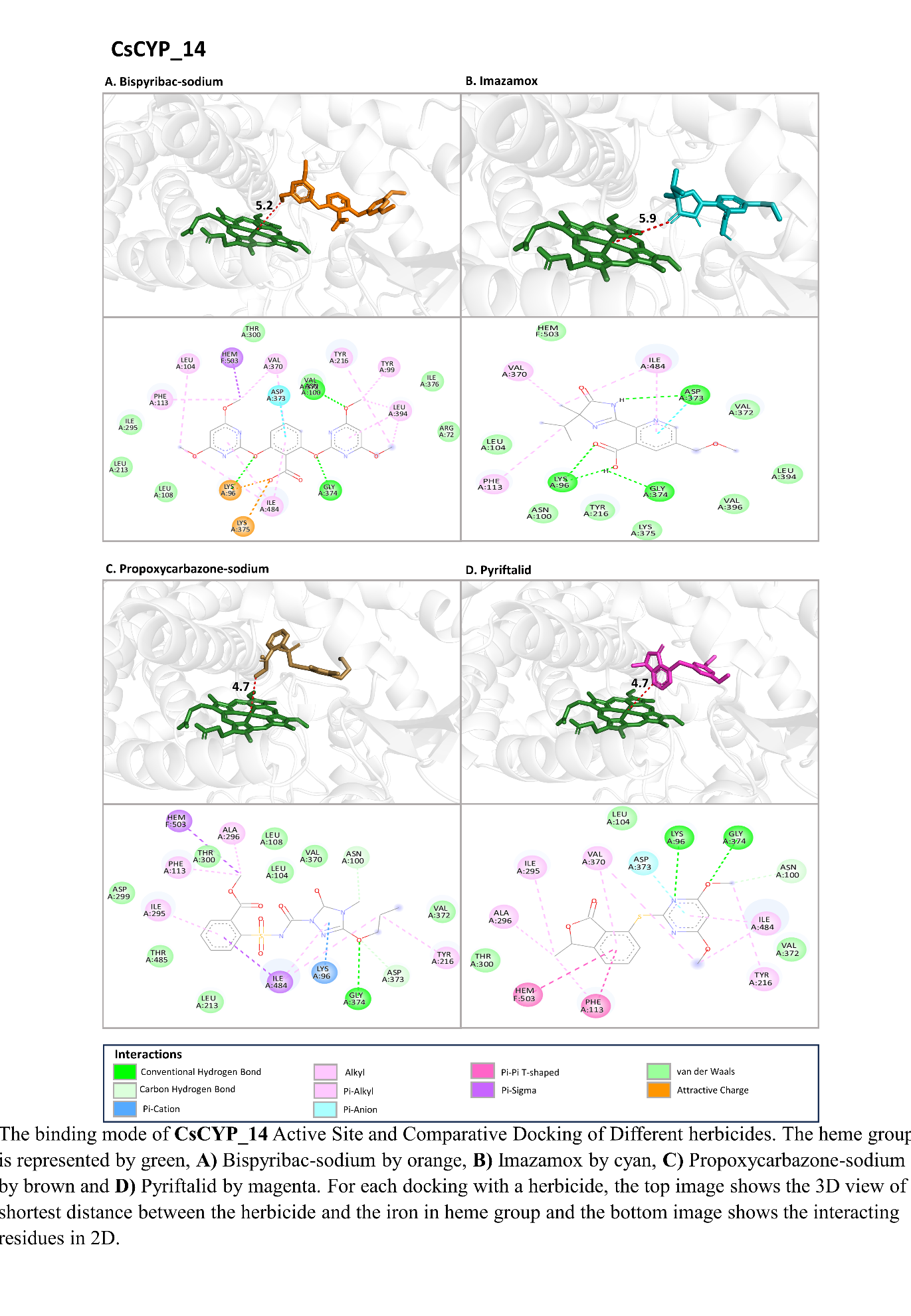


Figure S4: The binding mode of CsCYP_14 with A) Bispyribac-sodium, B) Imazamox, C) Propoxycarbazone-sodium and **D)** Pyriftalid at the active site. For each docking with a herbicide, the top image shows the 3D view of shortest distance between the herbicide and the iron in heme group and the bottom image shows the interacting residues in 2D.


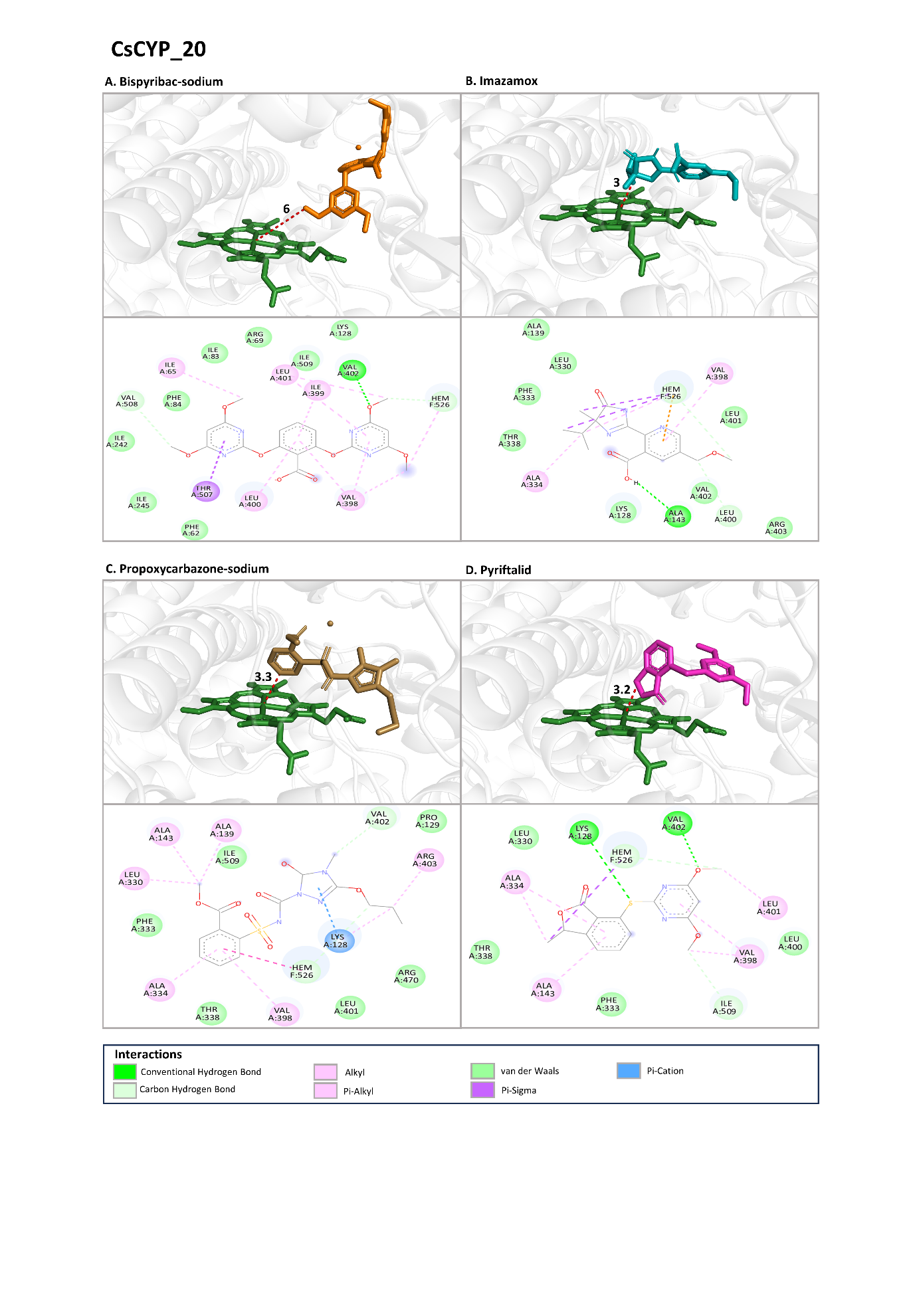


Figure S5: The binding mode of CsCYP_20 with A) Bispyribac-sodium, B) Imazamox, C) Propoxycarbazone-sodium and **D)** Pyriftalid at the active site. For each docking with a herbicide, the top image shows the 3D view of shortest distance between the herbicide and the iron in heme group and the bottom image shows the interacting residues in 2D.


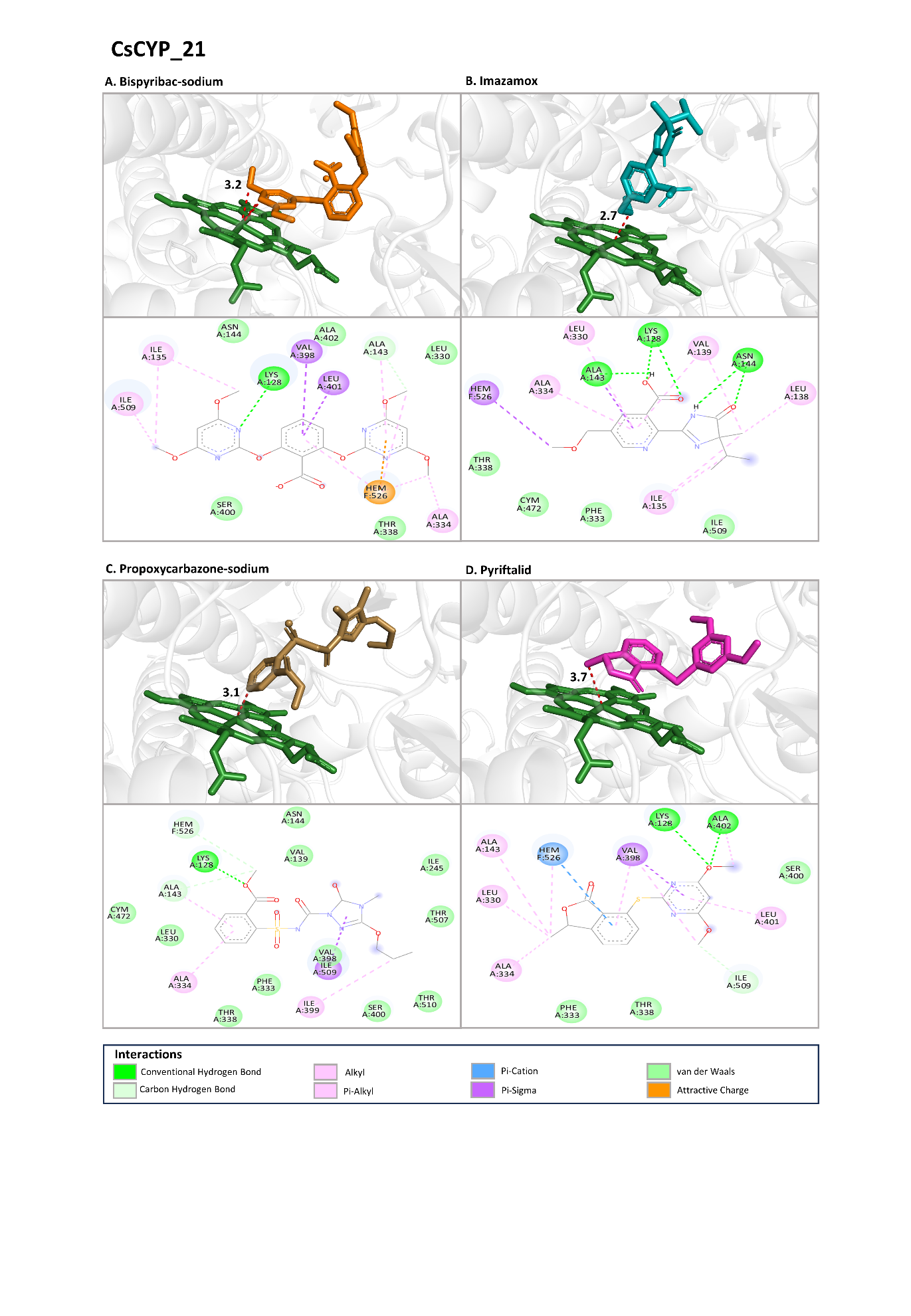


Figure S6: The binding mode of CsCYP_21 with A) Bispyribac-sodium, B) Imazamox, C) Propoxycarbazone-sodium and **D)** Pyriftalid at the active site. For each docking with a herbicide, the top image shows the 3D view of shortest distance between the herbicide and the iron in heme group and the bottom image shows the interacting residues in 2D.


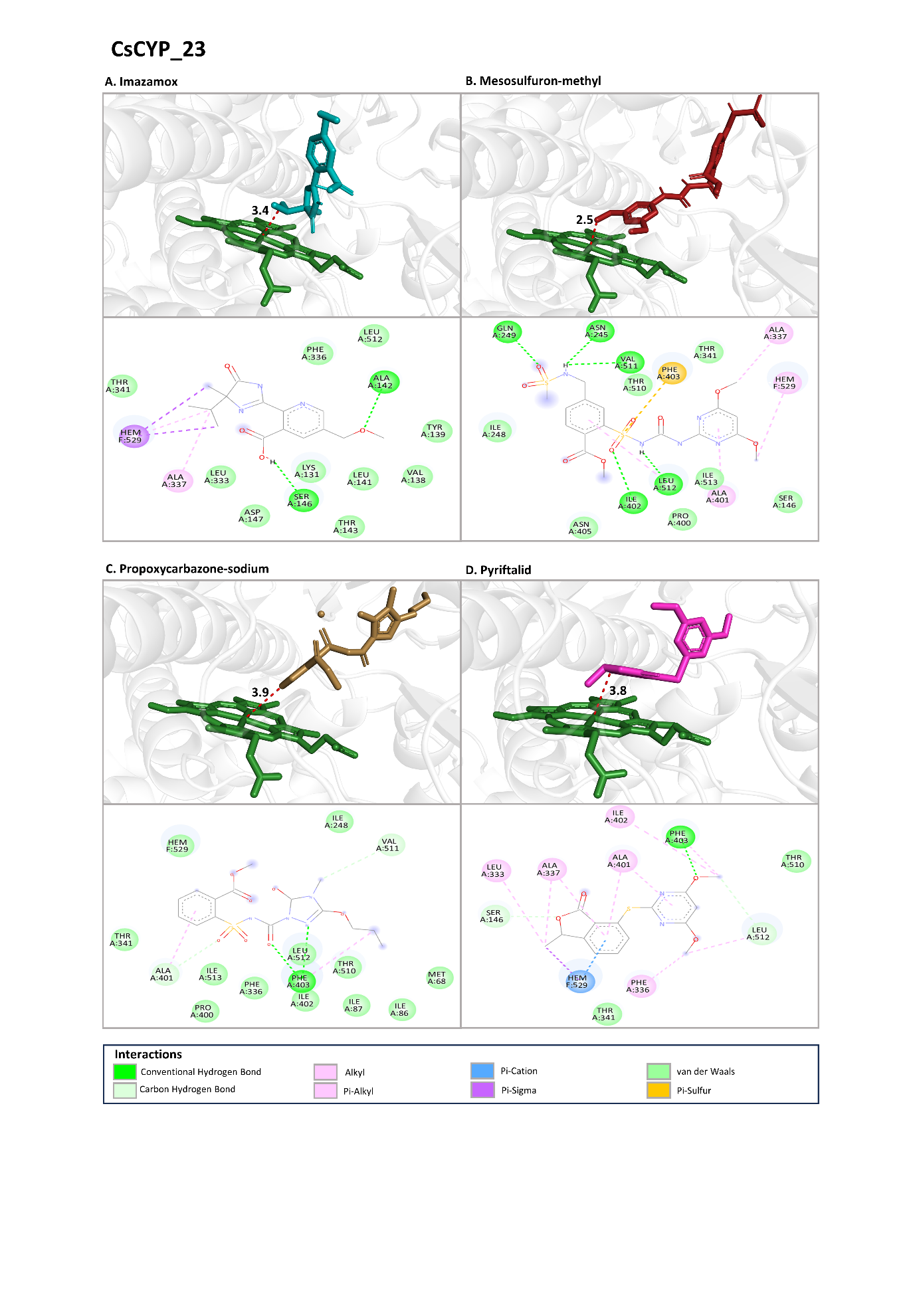


Figure S7: The binding mode of CsCYP_23 with A) Imazamox, B) Mesosulfuron-methyl, C) Propoxycarbazone-sodium and **D)** Pyriftalid at the active site. For each docking with a herbicide, the top image shows the 3D view of shortest distance between the herbicide and the iron in heme group and the bottom image shows the interacting residues in 2D.


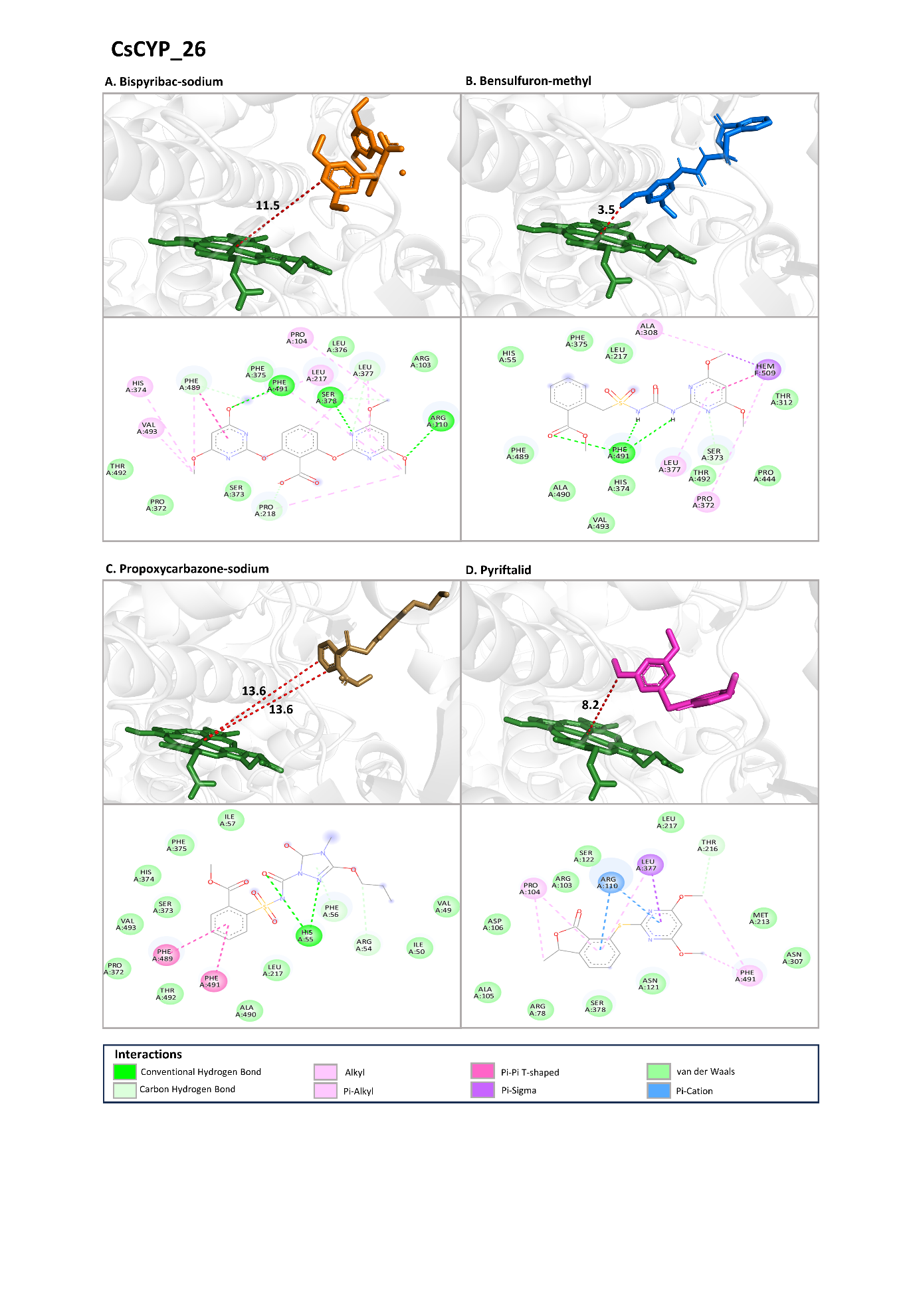


Figure S8: The binding mode of CsCYP_26 with A) Bispyribac-sodium, B) Bensulfuron-methyl, C) Propoxycarbazone-sodium and **D)** Pyriftalid at the active site. For each docking with a herbicide, the top image shows the 3D view of shortest distance between the herbicide and the iron in heme group and the bottom image shows the interacting residues in 2D.


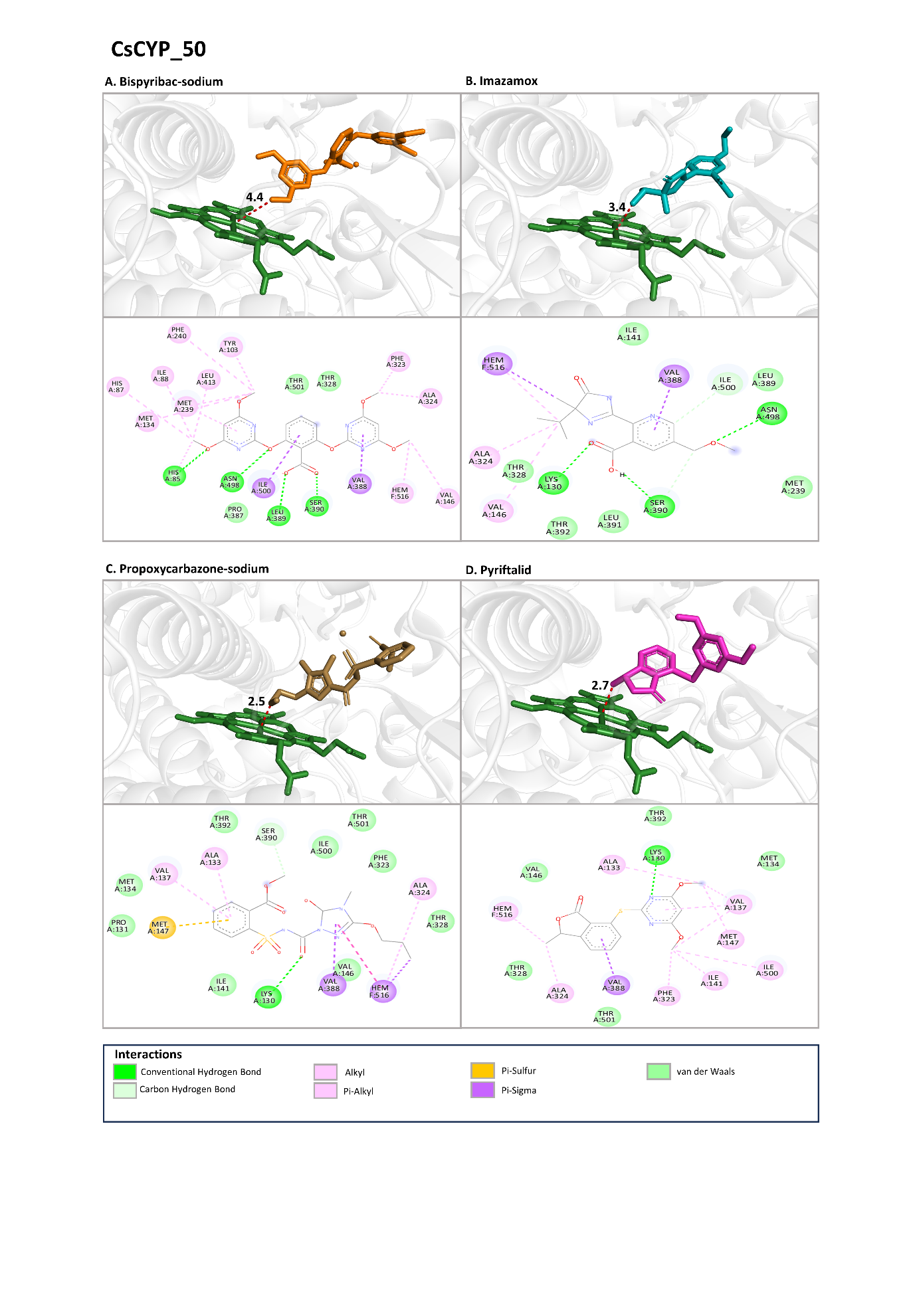


Figure S9: The binding mode of CsCYP_50 with A) Bispyribac-sodium, B) Imazamox, C) Propoxycarbazone-sodium and **D)** Pyriftalid at the active site. For each docking with a herbicide, the top image shows the 3D view of shortest distance between the herbicide and the iron in heme group and the bottom image shows the interacting residues in 2D.


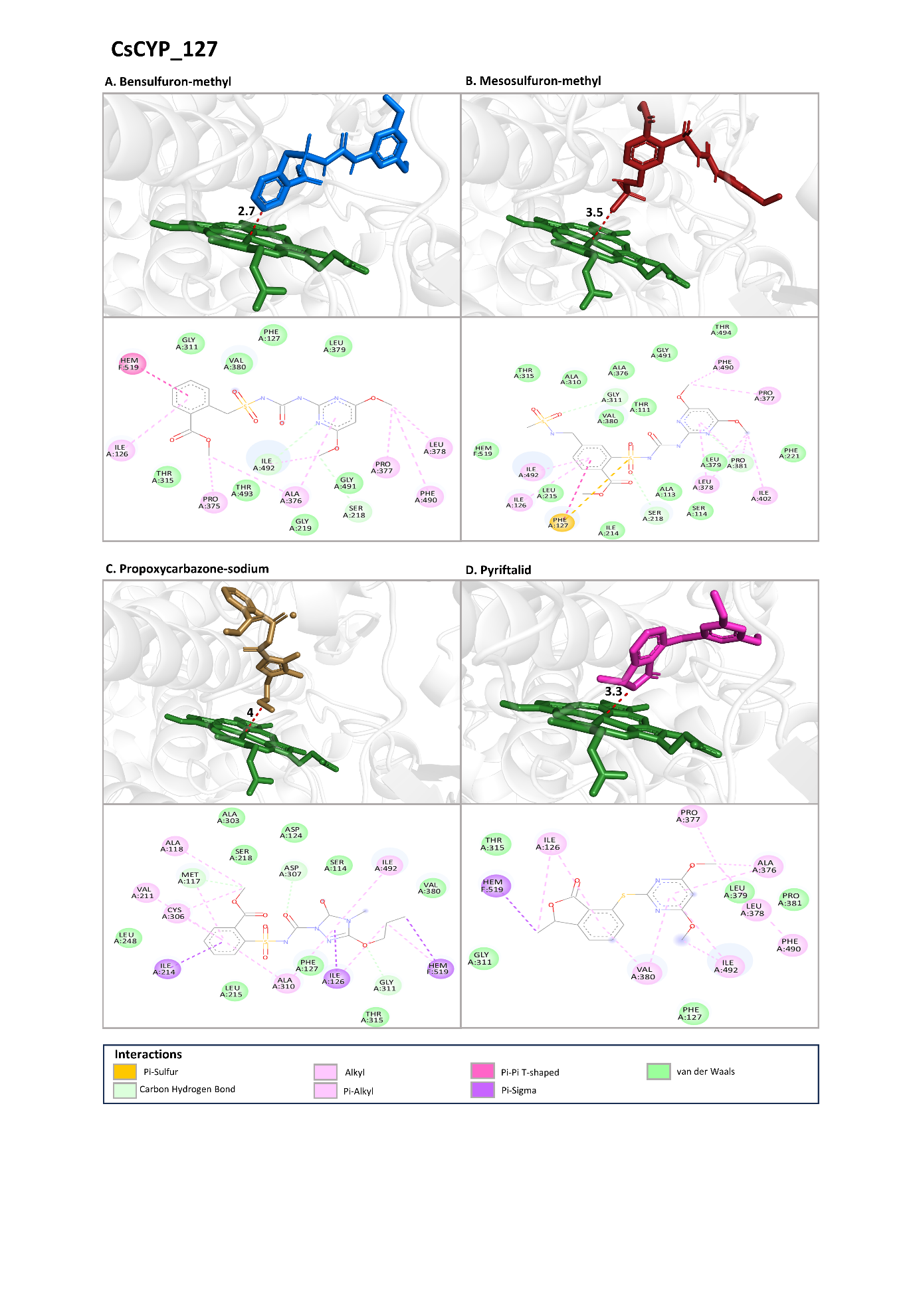


Figure S10: The binding mode of CsCYP_127 with A) Bensulfuron-methyl, B) Mesosulfuron-methyl, C) Propoxycarbazone-sodium and **D)** Pyriftalid at the active site. For each docking with a herbicide, the top image shows the 3D view of shortest distance between the herbicide and the iron in heme group and the bottom image shows the interacting residues in 2D.


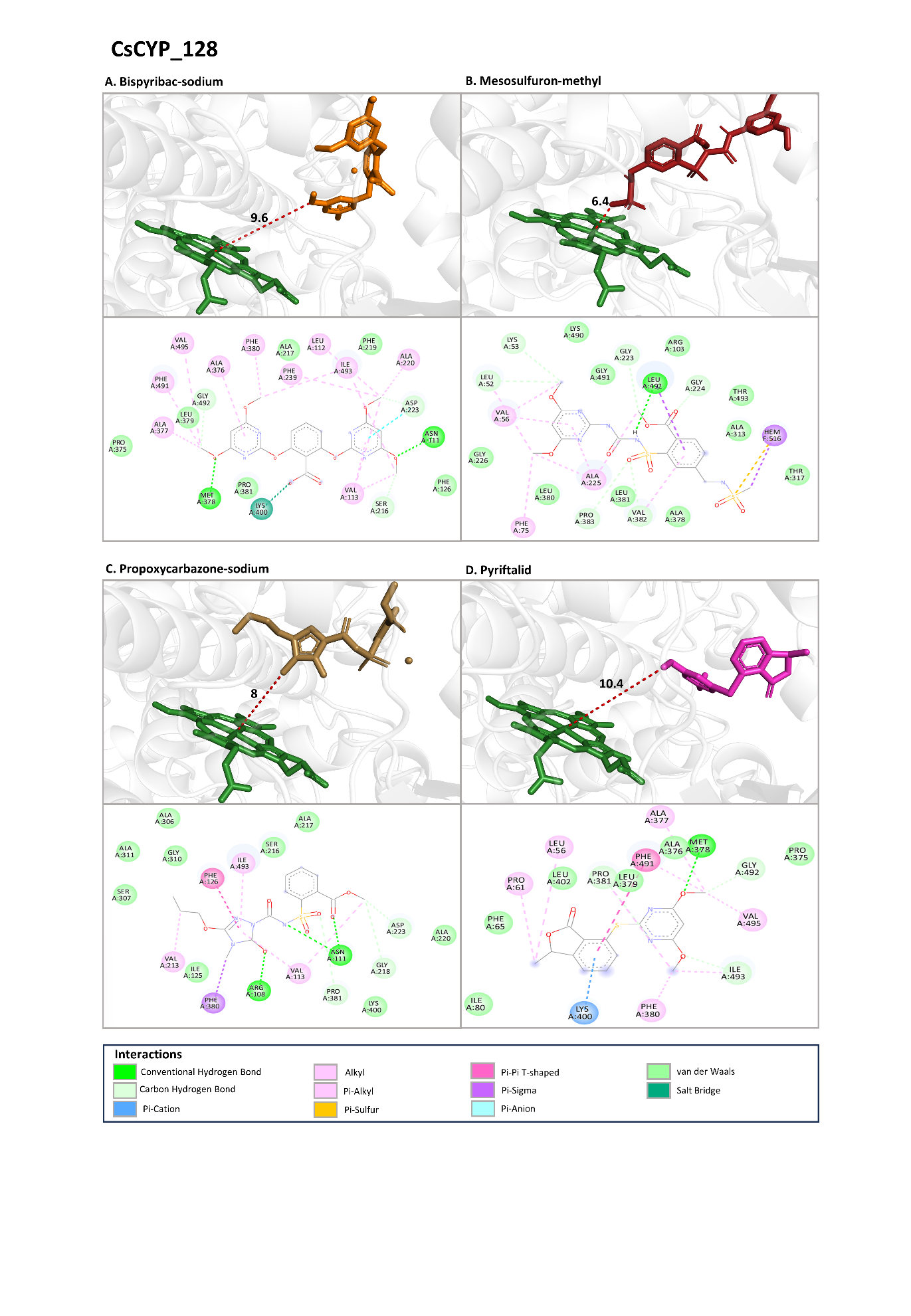


Figure S11: The binding mode of CsCYP_128 with A) Bispyribac-sodium, B) Mesosulfuron-methyl, C) Propoxycarbazone-sodium and **D)** Pyriftalid at the active site. For each docking with a herbicide, the top image shows the 3D view of shortest distance between the herbicide and the iron in heme group and the bottom image shows the interacting residues in 2D.


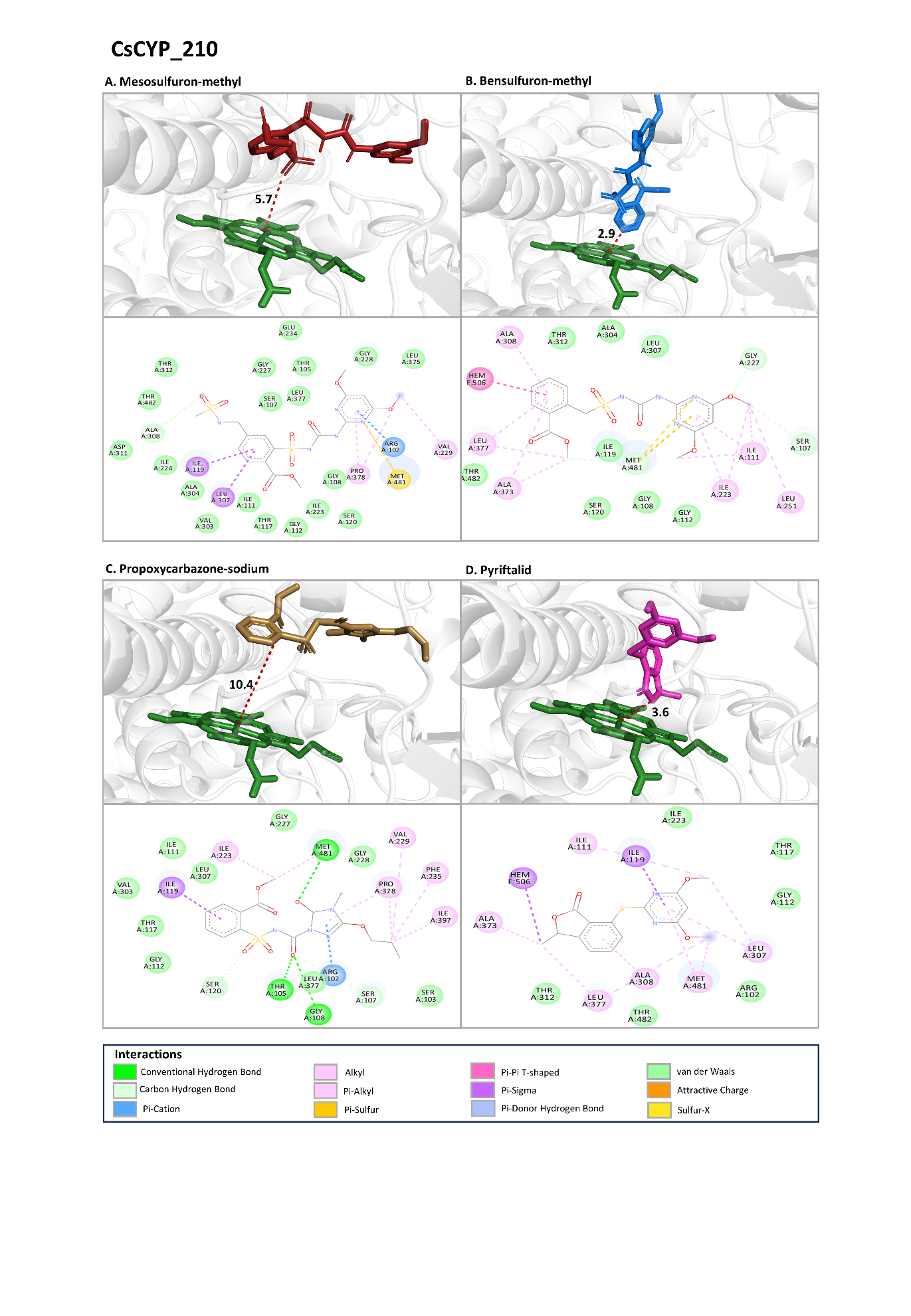


Figure S12: The binding mode of CsCYP_210 with A) Mesosulfuron-methyl, B) Bensulfuron-methyl, C) Propoxycarbazone-sodium and **D)** Pyriftalid at the active site. For each docking with a herbicide, the top image shows the 3D view of shortest distance between the herbicide and the iron in heme group and the bottom image shows the interacting residues in 2D.


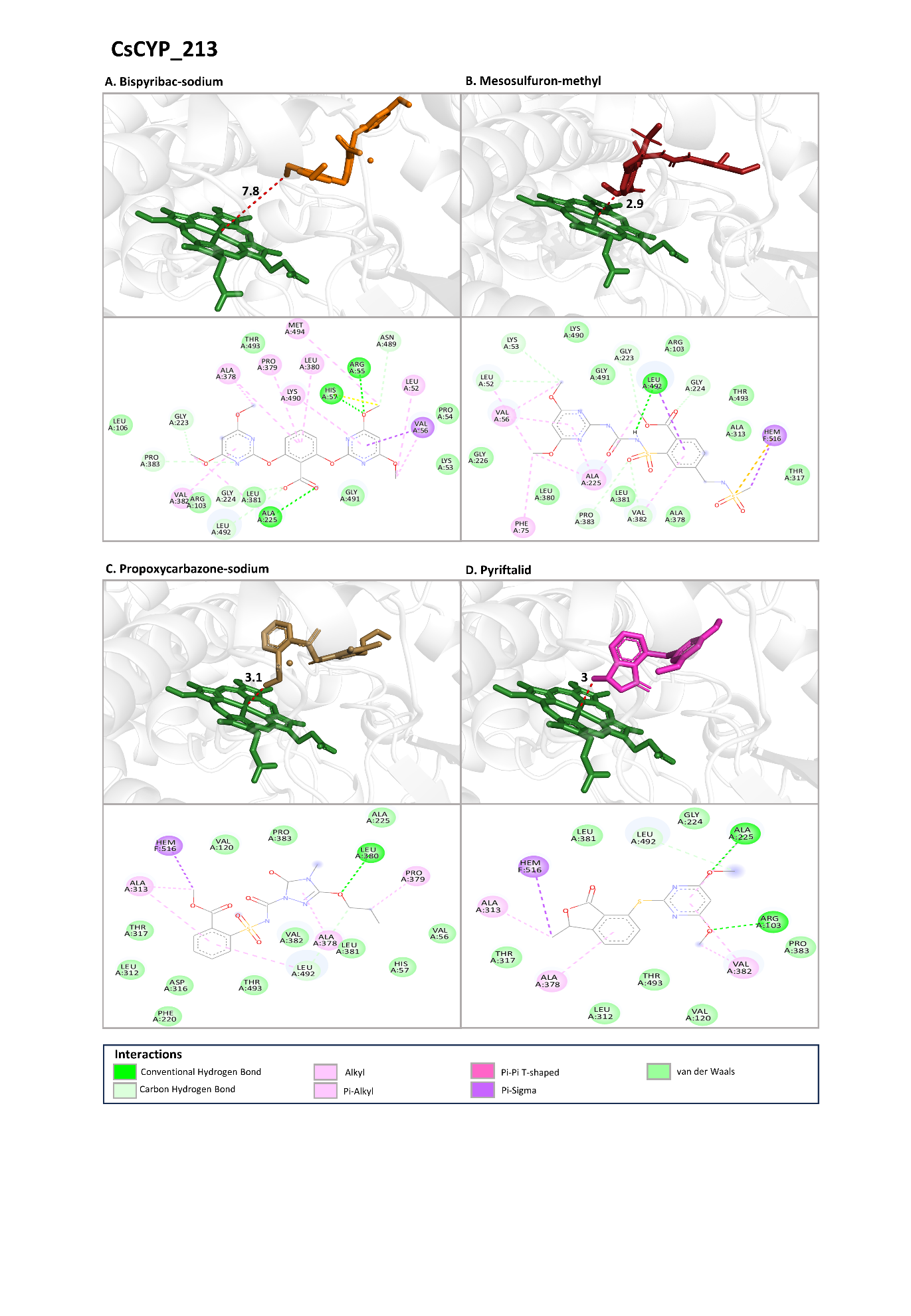


Figure S13: The binding mode of CsCYP_213 with A) Bispyribac-sodium, B) Mesosulfuron-methyl, C) Propoxycarbazone-sodium and **D)** Pyriftalid at the active site. For each docking with a herbicide, the top image shows the 3D view of shortest distance between the herbicide and the iron in heme group and the bottom image shows the interacting residues in 2D.


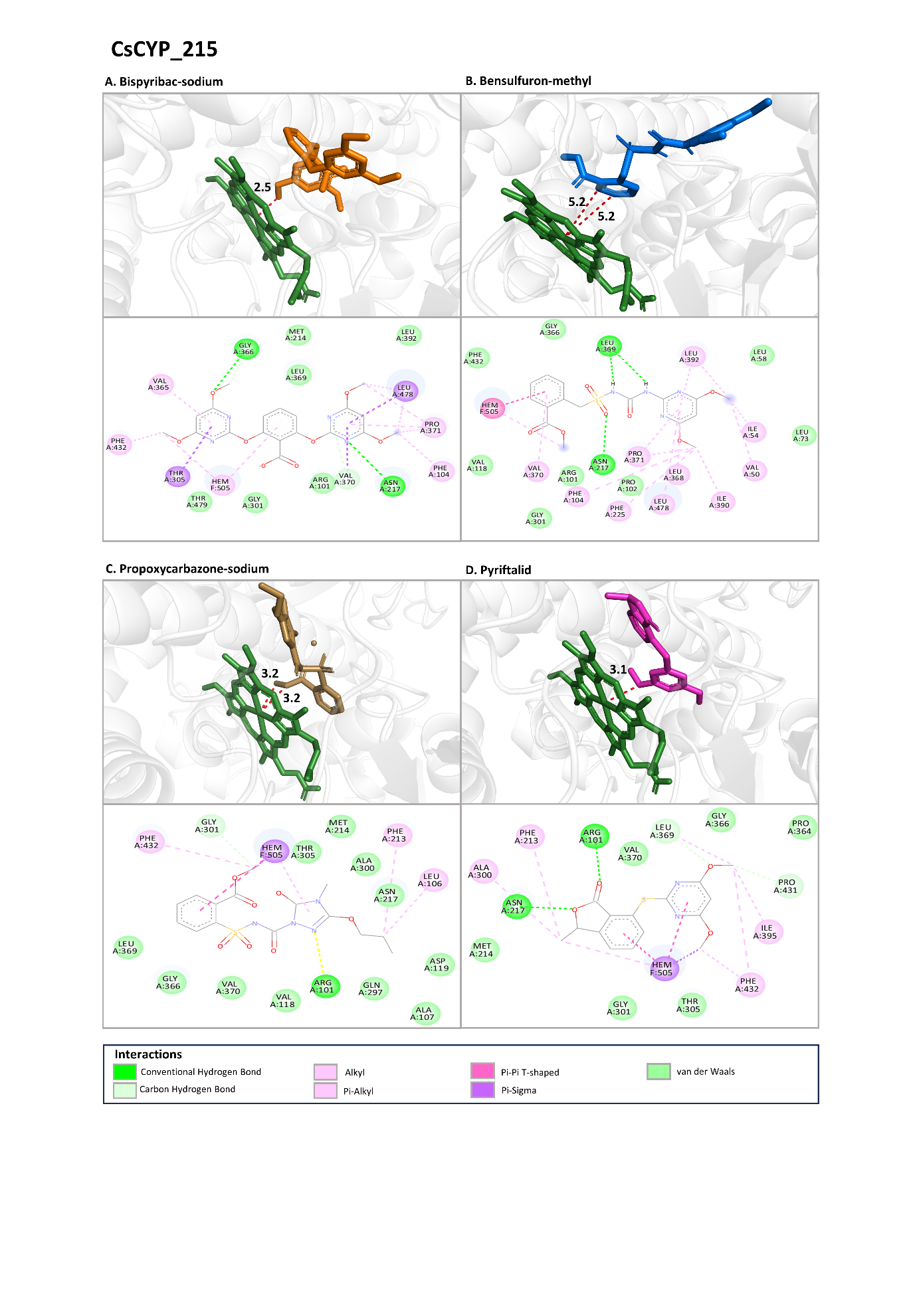


Figure S14: The binding mode of CsCYP_215 with A) Bispyribac-sodium, B) Bensulfuron-methyl, C) Propoxycarbazone-sodium and **D)** Pyriftalid at the active site. For each docking with a herbicide, the top image shows the 3D view of shortest distance between the herbicide and the iron in heme group and the bottom image shows the interacting residues in 2D.


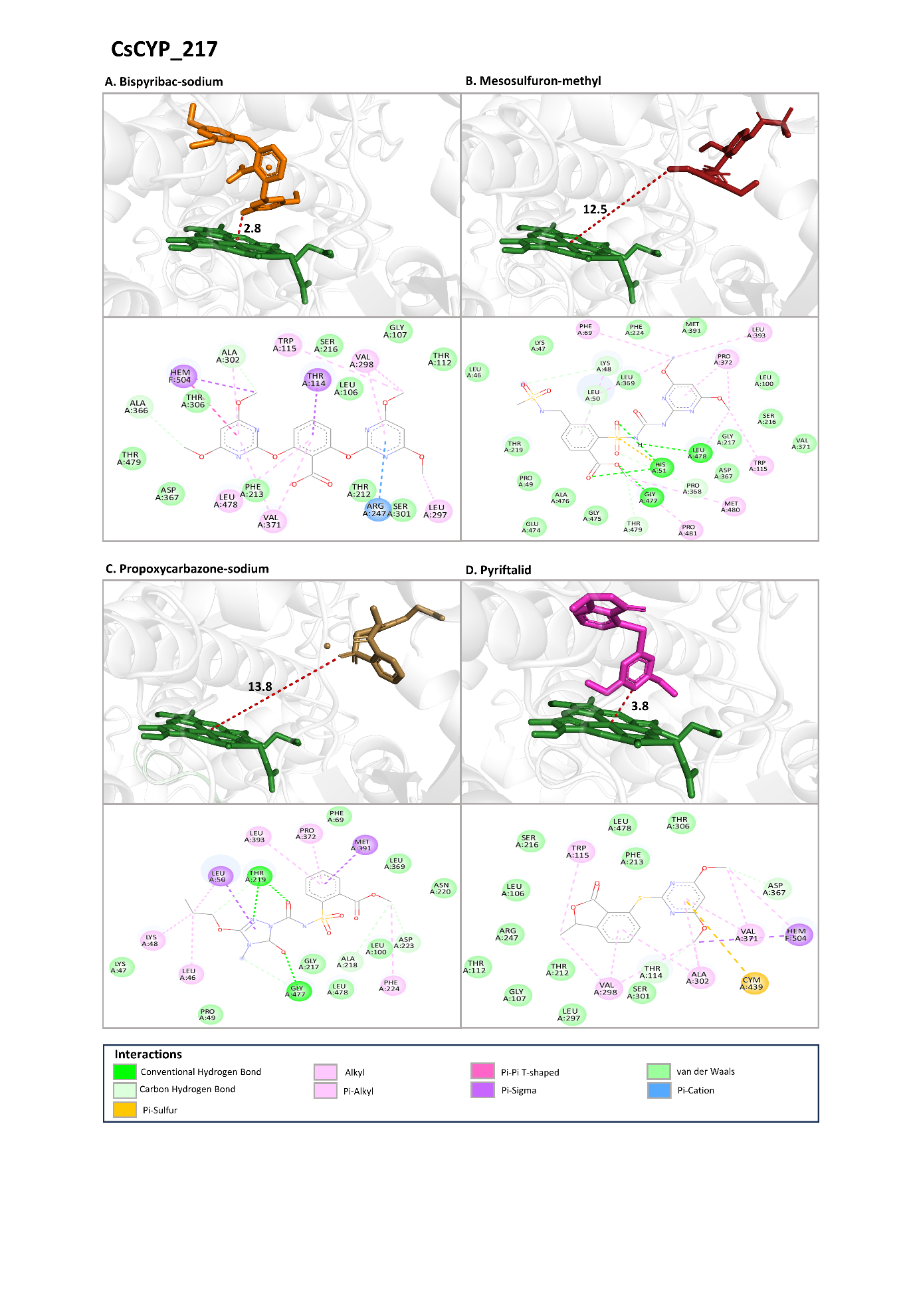


Figure S15: The binding mode of CsCYP_217 with A) Bispyribac-sodium, B) Mesosulfuron-methyl, C) Propoxycarbazone-sodium and **D)** Pyriftalid at the active site. For each docking with a herbicide, the top image shows the 3D view of shortest distance between the herbicide and the iron in heme group and the bottom image shows the interacting residues in 2D.


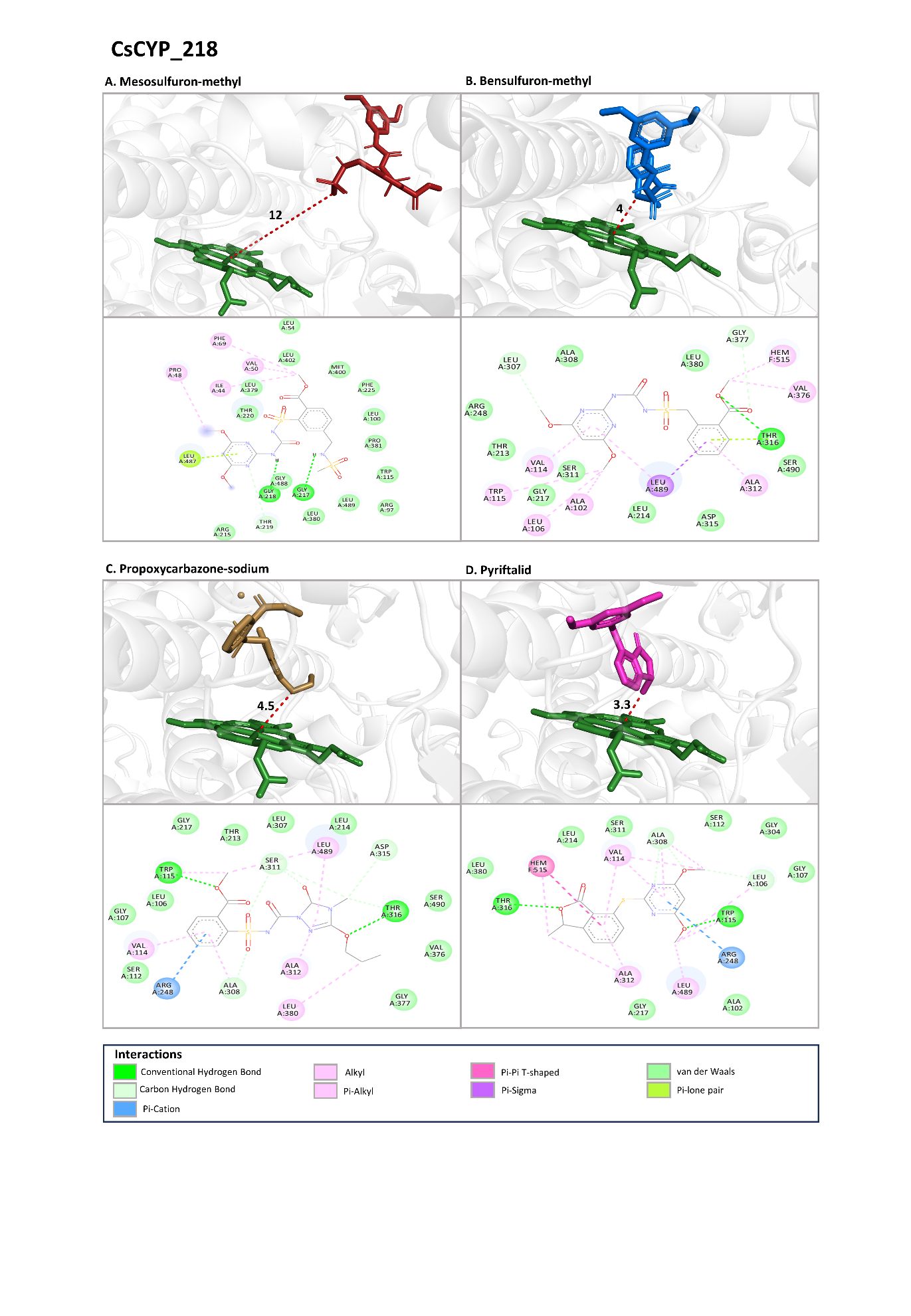


Figure S16: The binding mode of CsCYP_218 with A) Mesosulfuron-methyl, B) Bensulfuron-methyl, C) Propoxycarbazone-sodium and **D)** Pyriftalid at the active site. For each docking with a herbicide, the top image shows the 3D view of shortest distance between the herbicide and the iron in heme group and the bottom image shows the interacting residues in 2D.
